# Outcomes of intended temporary stomas in Crohn's disease (INTESTINE study): international, multicentre, retrospective study

**DOI:** 10.1093/bjsopen/zraf010

**Published:** 2025-06-02

**Authors:** Claire Perrott, Giacomo Calini, Alice Gori, Matteo Rottoli, Maria E Flacco, Lamberto Manzoli, Zoe Garoufalia, Steven D Wexner, Christos Kontovounisios, Muhammed Elhadi, Valerio Celentano, the INTESTINE Study Group, N Avellaneda, A Potolicchio, J P Muñoz, N Avellaneda, A S Abdelrahman, Sara Mansour Mostafa, N de' Angelis, C A Schena, F Marchegiani, M Kelm, S Flemming, J Lock, D Politis, P Ioannis, O Mangana, L Chardalias, Y Zager, N Horesh, G Calini, G Terrosu, L Martinuzzo, D Muschitiello, C Biddau, A Braini, F Tumminelli, A Gori, M Rottoli, S Cardelli, A Belvedere, C Isopi, G Gallo, M Trompetto, G Clerico, A Realis Luc, G Gallo, A Mingoli, P Lapolla, G Brachini, G Mazzarella, O Ghazouani, R Galleano, M Malerba, F Menegon Tasselli, G Pellino, G Rizzo, M Cappello, L Carrozza, G Mazzarella, I A Muttillo, E M Muttillo, B Picardi, N Bazzi, S Dbouk, Z Chaalan, M Bazzi, A Alkaseek, H Bileid Bakeer, H Shames, H Aboudlal, A Kredan, Q Qutaiba, A Y Abu Rumaila, J Q Al Safwan, N A Al Turki, A Bunyian, N Fernandes Montes, M Martí Gallostra, R Pintos Garza, V Vigorita, E Moncada Iribarren, I De Ariño-Hervas, I Aguirre-Allende, J M Enriquez-Navascués, M J Padilla-Otamendi, M Sanchez-Rodriguez, C Pérez-Carpio, P Tejedor, D Velayos Herraez, M D Cancelas Felgueras, M Estaire Gómez, E P Cagigal Ortega, D Plazas, M Millan, C Gutierrez, P Montalbán, F Blanco-Antona, A E Valera Montiel, R Kozan, S Leventoglu, H H Ceylan, S Ozaydin, E B Bostanci, T Colak, C Ozcan, I C Eray, E Aytaç, D Selvakumar, L Hancock, N Jabble, D Warrington, N Ahmed, T Hussain, J Cooper, A Gendia, J Ahmed, K Exarchou, N Eardley, B Davies, C A Manzo, V Celentano, S Pérez-Ajates, S Seth, K Sriskandarajah, T Chouari, E Matthews, R Bethune, M Abuelgasim, A Smith, E Brownson, G Nicholson, I Campbell, A Subramanian, A Tonsi, J Siby, Z Garoufalia, S D Wexner, P Zhou, R Gefen, M Arjonilla, F Monzur, M Al-Sadawi, N de’ Angelis, O Mangana, N Horesh, G Calini, M Elhadi, P Tejedor, V Vigorita, S Leventoglu, V Celentano, C Perrott, M Rottoli, V Celentano, G Calini, G Calini, C Kontovounisios, Z Garoufalia, M Elhadi, S D Wexner, S Blackwell, C Perrot, G Calini, A Gori

**Affiliations:** Inflammatory Bowel Disease and Ileoanal Pouch Surgery Centre, Chelsea and Westminster Hospital NHS Foundation Trust, London, UK; ClinicaChirurgica, University Hospital of Udine, Udine, Italy; Surgery of the Alimentary Tract, IRCCS Azienda Ospedaliero-Universitaria di Bologna, Bologna, Italy; Department of Medical and Surgical Sciences, Alma Mater Studiorum—University of Bologna, Bologna, Italy; Surgery of the Alimentary Tract, IRCCS Azienda Ospedaliero-Universitaria di Bologna, Bologna, Italy; Department of Medical and Surgical Sciences, Alma Mater Studiorum—University of Bologna, Bologna, Italy; Surgery of the Alimentary Tract, IRCCS Azienda Ospedaliero-Universitaria di Bologna, Bologna, Italy; Department of Medical and Surgical Sciences, Alma Mater Studiorum—University of Bologna, Bologna, Italy; Department of Environmental and Preventive Sciences, University of Ferrara, Ferrara, Italy; Department of Medical and Surgical Sciences, Alma Mater Studiorum—University of Bologna, Bologna, Italy; Cleveland Clinic Florida, Ellen Leifer Shulman and Steven Shulman Digestive Disease Center, Weston, Florida, USA; Cleveland Clinic Florida, Ellen Leifer Shulman and Steven Shulman Digestive Disease Center, Weston, Florida, USA; Inflammatory Bowel Disease and Ileoanal Pouch Surgery Centre, Chelsea and Westminster Hospital NHS Foundation Trust, London, UK; 2nd Surgical Department, Evaggelismos Athens General Hospital, Athens, Greece; Department of Surgery, The Royal Marsden NHS Foundation Trust, London, UK; Department of Surgery and Cancer, Imperial College, London, UK; Faculty of Medicine, Tripoli University Hospital, Tripoli, Libya; Inflammatory Bowel Disease and Ileoanal Pouch Surgery Centre, Chelsea and Westminster Hospital NHS Foundation Trust, London, UK; Department of Surgery and Cancer, Imperial College, London, UK

## Abstract

**Background:**

Patients with ileocolic Crohn’s disease often require surgery that can result in temporary stoma formation. Stomas are associated with a morbidity and can negatively impact quality of life. This study aimed to investigate the short-term (6-month) and mid-term (18-month) outcomes of intended temporary stomas in patients with Crohn’s disease.

**Methods:**

A trainee-led, international multicentre, retrospective study was conducted on all patients who underwent surgery for Crohn’s disease in collaborating centres over 4 years (2017–2020). The primary outcome was the proportion of patients with Crohn’s disease who underwent stoma reversal surgery by 6- and 18-month postoperative follow-up. Secondary outcomes included: the time interval between formation and reversal of stoma and predictors for non-reversal and stoma-related morbidity (postoperative complications, related readmissions and complications due to stoma reversal surgery).

**Results:**

A total of 401 patients underwent stoma formation for Crohn’s disease over the 4 years across the 44 collaborating centres. The temporary stomas had been reversed in 30.2% of patients at the 6-month and 56.9% at the 18-month follow-up. Reasons for non-reversal included ongoing medical treatment for Crohn’s disease (respectively 6-month and 18-month: 37.6%, 39.3%), patient unfit for surgery (respectively 6-month and 18-month: 14.5%, 16.8%), patient preference (respectively 6-month and 18-month: 12.1%, 20.2%) and due to waiting lists (respectively 6-month and 18-month: 12.1%, 8.1%). Overall, 63.3% of patients had a temporary stoma reversed with a median time interval of 6 months. The stoma-related overall morbidity rate was 29.4%.

**Conclusions:**

A large proportion of temporary stomas for Crohn’s disease were not reversed at 6 and 18 months following initial surgery. Patients are exposed to the risk of non-reversal and risk of developing stoma complications for significantly longer intervals of time and, in some cases, indefinitely.

## Teaser

This trainee-led, international, multicentre, retrospective study includes patients with Crohn’s disease performing a temporary stoma during surgery. The stoma reversal surgery rate at 6 and 18 months of postoperative follow-up is reported together with time interval between formation and reversal of the stoma, predictors for non-reversal and stoma-related morbidity.

## Introduction

The incidence and prevalence of inflammatory bowel diseases (IBD) such as Crohn’s disease (CD) and ulcerative colitis is increasing worldwide^1^. Europe has one of the highest prevalence rates, with 0.2% of the population affected by IBD^1^, and CD affects 115 000 patients in the UK^2^. Despite medical treatment, up to 80% of these patients will require surgical intervention during their lives^3^ for reasons such as medical refractory disease, perianal or enteric fistulae, perforation, obstruction or strictures^4^. Surgical intervention may require the formation of a stoma, which can be temporary or permanent, and stomas are associated with morbidity and can negatively impact quality of life, particularly when complications occur. Stoma-related complications include parastomal and incisional hernia, prolapse, stenosis, retraction, and prolonged and repeated hospital admissions due to high output. CD is a lifelong condition and can require multiple operations, exposing patients to the risk of complications at each procedure. Furthermore, this health burden will only rise with the increasing incidence and increased life expectancy globally.

Key performance indicators are a quality measurement technique initially used in the manufacturing industry to reduce variation in standards. Their use has been transferred to healthcare where standards of care can be monitored across a variety of healthcare settings. A Delphi Consensus study in 2017^5^ proposed KPIs for the surgical management of IBD, including measuring postoperative complications, rate of reintervention and readmission, and the timing and rate of defunctioning stoma reversal. Due to the morbidity and impact on quality of life of stomas, they should be reversed in a timely manner; however, no studies have investigated the outcome of intended temporary stomas in CD, whether planned reversal occurs, how long it occurs after formation and what the reasons are for non-reversal.

This study aimed to investigate outcomes of intended temporary stomas in patients with CD in the short-term (6-month) and long-term (18-month) follow-up intervals.

## Methods

### Study setting

This trainee-led, international multicentre, retrospective, observational study was designed and reported according to the STROBE guidelines^6^. All centres required dedicated IBD colorectal surgeons and gastroenterologists who held regular IBD multidisciplinary team (MDT) meetings. The study steering group consisted of patient representatives, surgical trainees, colorectal surgeons with expertise in CD surgery, and methodological leads with expertise in leading and developing multicentre studies. The Steering Group, the National Coordinators, and Collaborative Authors of The INTESTINE Study Group are reported in the *Supplementary materials*.

### Study participants

The study included patients who underwent the formation of a stoma for CD and met the inclusion criteria below, as previously published in the study protocol^7^.

- Inclusion criteria: patients aged 18 years or over; underwent an intended temporary stoma formation for CD; between January 2017 and December 2020, the 4-year study recruitment interval; follow-up of 18 months for each patient; either elective or urgent/emergency surgery. Included procedures: ileocolonic resection (right hemicolectomy, extended right hemicolectomy, ileocaecal resection, redo ileocolic resection); segmental colonic resections including subtotal colectomy, left hemicolectomy, transverse colectomy, anterior resection, Hartmann’s procedure; small bowel resection, strictureplasty as a sole procedure; formation of ileostomy or colostomy as a sole procedure for the treatment of complex ileocolic disease or perianal disease. Patients were excluded if they underwent the following procedures: panproctocolectomy, proctectomy.

### Outcomes of interest

The primary outcome was the proportion of patients with CD who underwent stoma reversal surgery at 6- and 18-month postoperative follow-up.

- Secondary outcomes included: time interval between the stoma formation and reversal; predictors for no/late (more than 18 months) reversal and for reversal ≤6 months; morbidity related to stoma presence and reversal, which includes stoma-related postoperative complications, 6-month or 18-month stoma-related readmissions, and complications due to stoma reversal surgery.

Subgroup analysis was carried out for patients who underwent stoma formation in the context of colonic, rectal and perianal CD. In this subset of patients, we explored the time interval between the stoma formation and reversal and predictors for no/late (>18 months) reversal and reversal ≤6 months.

An accessory evaluation was carried out to investigate the impact of the coronavirus disease (COVID)-19 pandemic on the primary outcome and on the time interval between the stoma formation and reversal. October 2019 was chosen as a threshold to investigate the impact of the COVID-19 pandemic on the time interval between stoma formation and reversal. As March 2020 was the time of initial disruption of healthcare in the centres included in this study, we anticipated 6 months to include the majority of patients still having a stoma and so likely being affected by the COVID-19 pandemic. Pre-COVID includes patients who underwent stoma formation before October 2019. The post-COVID group included October 2019 and the following months.

Descriptions of the sample size calculation, study procedures, authorship, ethical considerations and data storage have already been published in the protocol^7^.

### Data collection

Data collection was carried out from 1 October 2022 to 31 December 2022. This was then extended by 4 weeks to 31 January 2023 (*Supplementary material*, *Fig. S1*). Each centre reviewed its clinical records, including databases, operating theatre registries, IBD MDT registries and clinical coding searches, to identify patients who had undergone surgery for CD with stoma formation. The identified patients were then screened against the inclusion criteria, and the following data was collected: demographic data, disease-specific and surgery-specific information, and surgical outcomes. Further information is reported in the study protocol^7^.

### Data verification and validation

Each centre nominated an independent data validator who did not participate in the initial data collection. The data validator reviewed 20% of the patients who were uploaded by their centre. Data validation ran for 2 weeks from 20 March 2023.

In addition to data validation, a process of database revision and data cleansing (data verification) continued from 1 January 2023 to 1 September 2023 (*Supplementary material, Fig. S1*).

The database was screened for inclusion and exclusion criteria, missing or incongruent data (for example date of reversal before the date of primary surgery) and absent follow-ups. Also, collaborating centres were asked to confirm outliers in the variables of greater interest (for example stoma formation and reversal dates), and related to the subgroup analyses (for example CD localization, COVID pandemic impact).

Collaborating centres were emailed and the collaborating centre’s validator was asked to review missing or incongruent data. Centres were emailed a minimum of three times in a 1-month window. The time given for data verification was based on data amount and collaborating centres’ request on a case-by-case scenario. Patients with unresolved incongruency by unresponsive centres at the end of the data verification interval (*Supplementary material*, *Fig. S1*) were excluded.

### Statistical analysis

The differences in each recorded variable according to the surgery outcome (stoma reversal within 6 months, stoma reversal between 6 to 18 months, no or late (>18 months) stoma reversal) were initially examined using the chi-square test for categorical variables and *t*-test or Kruskal–Wallis test for normally distributed and non-normally distributed continuous variables respectively. Cox proportional hazards analysis was then used to compute the adjusted relative hazards of late or non-reversal by each variable, both in the total sample and in the subset of individuals with colonic, rectal or perianal CD location. In both models, the dependent variable was dichotomized into late/non-reversal *versus* stoma reversal within 18 months. Covariates were selected for inclusion in final models using a stepwise forward process with the following inclusion criteria: *P* < 0.150 at univariate analysis and ≥20% change in the hazard ratio of significant predictors. The variables: age, CD location, CD behaviour, parenteral nutrition prior to surgery, type of resection and type of stoma, were forced to enter the model. Schoenfeld’s test was carried out to check the validity of the proportional hazards assumption. Kaplan–Meier time-to-event curves were made, and log-ranks were used as appropriate to present time-to-stoma reversal in the total sample, in the individuals with colonic, rectal or perianal CD location (subgroup analysis), and to compare the impact of the COVID-19 pandemic (accessory evaluation). There were <5% missing values, thus no missing imputation technique was adopted. A *P* value of <0.050 was considered significant for all analyses, which were carried out using Stata, v. 13.1 (Stata Corp., College Station, TX, USA, 2013; http://www.stata.com/) and using R version 4.3.1 (RStudio Team (2020). RStudio: Integrated Development for R. RStudio, PBC, Boston, MA, USA; http://www.rstudio.com/).

## Results

Out of 426 patients collected, 25 patients were excluded for not meeting the inclusion criteria at data verification (*Supplementary material*, *Fig. S2*). Reasons for exclusion were: inadequate data about stoma formation and reversal by unresponsive centres (10), no stoma performed (10), panproctocolectomy (2), empty record ID (2) and surgery date outside the study interval (1). Overall, the study included complete follow-up data for 401 patients who underwent surgery with intended temporary stoma formation for CD between January 2017 and December 2020. Forty-four international centres with regular IBD MDT and dedicated IBD colorectal surgeons and gastroenterologists participated in The INTESTINE (INtended TEmporary STomas in CrohN’s disease) Study (*Supplementary material*, *Fig. S3*). Overall characteristics of the study participants and surgical characteristics and outcomes are reported in *Table 1* and *Table 2* respectively.

**Table 1:** Overall characteristics of the study participants

	Overall sample
Variables	(*n* = 401)
**Demographic and clinical characteristics**	
Age (years), mean(s.d.)	42.1(16.3)
Male sex	53.6
BMI (kg/m^2^), mean(s.d.)	23.5(5.8)
Familial IBD	10.5
**Risk factors and co-morbidities**	
At least one coexisting condition	35.7 (*n* = 143)*,†
Hypertension	37.8
Cardiovascular disease	17.5
Pulmonary disease	16.8
Neurological disease	7.0
Diabetes	19.6
Kidney disease	5.6
Ophthalmologic disease	2.1
Dermatologic disease	11.9
Osteoporosis	9.8
Other co-morbidities	30.8
**Smoking status**	
Never smoker	55.4
Past smoker	18.2
Current smoker	26.4 (*n* = 313)
Age at IBD diagnosis (years), mean(s.d.)	31.9(14.4)
**IBD location**	
Terminal ileum	27.2
Colon	14.5
Ileum	7.7
Rectum	0.7
Upper GI tract	0.7
Perianal	0.3
Others	0.3
≥ 2 concomitant locations	48.6
Colonic, rectal and perianal CD location	57.9
**CD behaviour**	
Stricturing	38.2
Penetrating	51.6
Other‡	10.2
**CD presentation at the time of surgery**	
Primary	51.5
Recurrent	48.5
**Medical treatment and laboratory assessment before surgery**	
Previous surgery due to CD	29.3
Preoperative medical treatment	90.7
**Type of medical treatment**†	(*n* = 361)
Biologics§	60.7
Antibiotics	59.6
Steroids	40.6
Azathioprine	18.1
Mercaptopurine	3.1
Cyclosporine	1.7
Thalidomide	1.1 (*n* = 320)
Albumin at surgery (g/dl), median (i.q.r.)	3.8 (3.1–5.2) (*n* = 343)
Haemoglobin at surgery (g/dl), median (i.q.r.)	11.7 (10.3–13.0) (*n* = 262)
CRP at surgery (mg/dl), median (i.q.r.)	7.7 (1.8–28.0)

Values are % unless otherwise indicated. *Based upon 143 observations. †More than one answer possible. ‡Including CD with non-stricturing, non-penetrating behaviour (*n* = 11), stricturing-perianal disease (*n* = 27), other undefined cases (*n* = 3). §Including Infliximab, Adalimumab, Vedolizumab, Ustekinumab or others. s.d., standard deviation; i.q.r., interquartile range; CD, Crohn’s disease; IBD, inflammatory bowel disease; GI, gastrointestinal; CRP, C-reactive protein.

**Table 2:** Surgical characteristics and outcomes of the study

	Overall sample
Variables	(*n* = 401)
**Main procedure—surgical approach**	
**ASA score class at surgery**	
I	6.3
II	63.2
III	28.5
IV	2.0
Emergency surgery	37.2
Parenteral nutrition preceding surgery	12.3
**Surgery indication**	
Perforation	7.2
Abscess	3.5
Bleeding	1.8
Obstruction	28.7
Fistula	7.2
Cancer	0.3
Persistent inflammation	11.0
2 coexisting indications	34.2
≥ 3 coexisting indications	6.2
**Type of intervention**	
Ileocaecal resection	31.4
Redo ileocolic resection	6.5
Small bowel resection	5.2
Subtotal colectomy	16.7
Segmental colectomy	8.0
Stoma formation only	10.2
≥ 2 surgical procedures	22.0
Laparoscopic surgery	30.4
**Timing of stoma formation**	
During index surgery	92.0
During re-intervention for complications	8.0
**Stoma location**	
Ileostomy	85.2
Colostomy	8.8
Ileocolostomy	4.7
Other	1.3
**Stoma configuration**	
Loop/loop-end	53.9
End	43.6
Other*	2.5
**Main procedure—outcomes**	
ICU admission	21.8
Duration of in-hospital stay (days), median (i.q.r.)	8 (6–14)
**30-day postoperative complications (Clavien–Dindo)**†	
0	45.4
I	16.5
II	20.0
IIIa	5.2
IIIb	11.2
IV–V	1.7
Medical complications‡	5.5
Surgical complications	29.2
**Type of surgical complication**§	(*n* = 117)
Procedure related	9.4
Stoma related	13.7
Anastomotic leak	23.1
Intra-abdominal collection	23.9
Postoperative ileum	19.7
Deep venous thrombosis	1.7
Wound infection	30.8
Others	7.7
Required reoperation	12.0
30-day mortality rate	0.8
**Stoma reversal surgery—procedures and outcomes**	
Stoma-related overall morbidity rate#	29.4
Overall stoma reversal	63.3
**Time between stoma formation and reversal**	(*n* = 254)
Within 6 months after formation	47.6
Between 6 and 18 months after formation	42.1
> 18 months after formation	10.3
**Type of surgery for stoma reversal**	(*n* = 254)
Open—midline laparotomy	31.9
Open—peristomal incision	62.6
Laparoscopy	5.5
**Type of procedure performed**	(*n* = 254)
Small bowel anastomosis	47.4
Ileocolic anastomosis	46.3
Colonic anastomosis	3.9
Other	2.4
New stoma creation at reversal surgery	2.0
Duration of in-hospital stay (days), median (i.q.r.)	5 (4–7)
**30-day postoperative complications (Clavien–Dindo)**†	
0	69.2
I	13.4
II	11.0
IIIa	2.4
IIIb	2.8
IV–V	1.2
Medical complications	1.2
Surgical complications	16.9
Required reoperation	3.2
Redo stoma at reoperation	1.6
30-day mortality rate	0.8
**6-month follow-up**	(*n* = 399)
6-month mortality rate	1.0
**Reason for stoma not being reversed**§	(*n* = 282)
Patient’s preference	12.1
Patient unfit for surgery	14.5
Ongoing medical treatment for CD	37.6
Waiting list	12.1
Low priority during COVID-19 pandemic	2.5
Others	13.8
**18-month follow-up**	(*n* = 389)
18-month mortality rate	2.1
**Reason for stoma not being reversed**§	(*n* = 173)
Patient’s preference	20.2
Patient unfit for surgery	16.8
Ongoing medical treatment for CD	39.3
Waiting list	8.1
Low priority during COVID-19 pandemic	2.3
Others	17.9

Values are % unless otherwise indicated. *Including continent ileostomy (Kock pouch, Barnett continent intestinal reservoir), mucus fistula. †Clavien–Dindo classification. ‡Including ≥1 among: pulmonary embolism, acute myocardial infarction, prolonged hypertension, prolonged hypotension, pneumothorax, pneumonia, vascular injury due to venous catheter. §More than one answer possible. #Including ≥1 among stoma formation-related postoperative complications (*n* = 16), 6-month (*n* = 24) or 18-month (*n* = 7) stoma-related readmissions, complications due to stoma reversal surgery (*n* = 80). ASA, American Society of Anesthesiologists; ICU, intensive care unit; CD, Crohn’s disease; COVID, coronavirus disease; s.d., standard deviation; i.q.r., interquartile range.

### Primary outcome

At the 6-month follow-up, 121 of 401 (30.2%) patients had the temporary stoma reversed, while 228 of 401 (56.9%) had it reversed at the 18-month follow-up.

The major reasons for stoma non-reversal at 6- and 18-month follow-ups were ongoing medical treatment for CD (respectively at 6- and 18-month follow-ups: 37.6% and 39.3%), patients unfit for surgery (respectively at 6- and 18-month follow-ups: 14.5% and 16.8%), patient’s preference (respectively at 6- and 18-month follow-ups: 12.1%, 20.2%), waiting list (respectively at 6- and 18-month follow-ups: 12.1% and 8.1%) (*Table 2*).

### Secondary outcomes

**Fig. 1:**
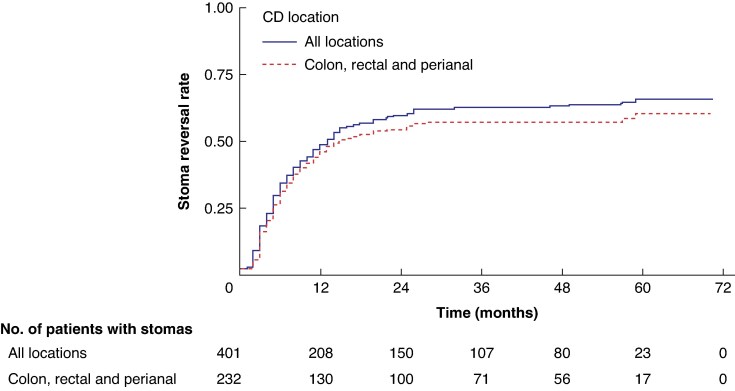
Time-to-event analysis between stoma formation and reversal in patients with Crohn’s disease (CD) All locations also includes colon, rectal and perianal CD locations.

The median follow-up was 45 months (i.q.r. 32–57). Overall, 254 of 401 (63.3%) patients had a temporary stoma reversed with a median time interval between the stoma formation and reversal of 6 months (i.q.r. 3–12). *Figure 1* shows the time-to-event analysis between stoma formation and reversal during the overall follow-up of the study.

To explore possible predictors of stoma reversal time intervals, patients were divided into three groups according to their stoma reversal status at the time of follow-ups: 121 patients in the ≤6 months, 107 in the 6–18 months and 173 in the late/non-reversal group (>18 months). *Table 3* reports a comparison of clinical and surgical characteristics among the three groups.

**Table 3:** Clinical and surgical characteristics of the sample, by time of stoma reversal

	No/late reversal*	*P*	Reversal ≤ 6 months	*P*	Reversal after 6–18 months	*P*†
Variables	(*n* = 173)		(*n* = 121)		(*n* = 107)	
Age (years), mean(s.d.)	44.6(17.2)	0.2	41.0(15.6)	0.06	39.5(15.2)	0.9
Male sex	46.2	0.30	52.1	0.001	67.3	0.02
BMI (kg/m^2^), mean(s.d.)	23.8(7.0)	0.8	23.1(4.2)	0.9	23.3(4.7)	0.9
Familial IBD	9.8	0.3	14.1	0.5	7.5	0.11
At least one risk factor or co-morbidity¶	43.3	0.012	28.9	0.037	30.8	0.4
**Smoking status**						
Never smoker	58.4	0.10	48.8	0.9	47.9	0.2
Past smoker	19.7	0.5	23.1	0.007	10.3	0.010
Current smoker	21.9 (*n* = 124)	0.2	28.1 (*n* = 102)	0.5	31.8 (*n* = 87)	0.048
Age at IBD diagnosis (years), mean(s.d.)	33.5(15.5)	0.2	30.0(12.5)	0.9	31.8(14.6)	0.9
Colonic, rectal and perianal CD location	73.4	<0.001	38.0	0.002	55.1	0.001
**CD behaviour**						
Stricturing	35.8	0.2	43.8	0.9	35.5	0.2
Penetrating	49.7	0.9	50.4	0.3	56.1	0.4
Others**	14.5	0.02	5.8	0.13	8.4	0.4
Recurrent CD presentation at the time of surgery	55.8	0.007	39.7	0.14	46.7	0.3
**Medical treatment and laboratory assessment before surgery**						
Previous surgery due to CD	39.3	0.02	25.8	0.001	16.8	0.09
Preoperative medical treatment	90.7 (*n* = 156)	0.4	93.4 (*n* = 113)	0.4	87.6 (*n* = 92)	0.13
						
Treatment with biologics††	68.6	0.003	51.3	0.09	58.7	0.3
Treatment with steroids	50.0	0.02	36.3	<0.001	29.7	0.3
	(*n* = 132)		(*n* = 110)		(*n* = 78)	
Albumin at surgery (g/dl), median (i.q.r.)	3.8 (3.0–23.7)	0.2	3.9 (3.3–4.7)	0.2	3.8 (3.1–4.5)	0.9
	(*n* = 141)		(*n* = 113)		(*n* = 89)	
Haemoglobin at surgery (g/dl), median (i.q.r.)	11.4 (10.0–12.8) (*n* = 111)	0.5	11.9 (10.7–13.2) (*n* = 87)	0.5	11.6 (10.7–13.0) (*n* = 64)	0.9
CRP at surgery (mg/dl), median (i.q.r.)	8.7 (2.2–33)	0.9	7.0 (1.6–28)	0.9	6.8 (1.9–20.9)	0.9
**Main procedure—surgical approach**						
**ASA score class at surgery**						
I	4.6	0.01	12.5	0.2	1.9	0.003
II	60.7	0.8	62.5	0.2	68.2	0.4
III	31.2	0.2	24.2	0.7	29.0	0.4
IV	3.5	0.14	0.8	0.2	0.9	0.9
Emergency surgery	37.6	0.5	33.9	0.7	40.2	0.3
Parenteral nutrition preceding surgery	16.8	0.10	10.0	0.03	7.5	0.5
**Surgery indication**						
Perforation	5.8	0.6	7.4	0.3	9.4	0.6
Obstruction	27.2	0.09	36.4	0.4	22.4	0.06
Persistent inflammation	15.0	0.2	9.9	0.02	5.6	0.2
Others‡‡	13.9	0.2	9.1	0.8	14.9	0.2
≥ 2 coexisting indications	38.1	0.9	37.2	0.11	47.7	0.11
**Type of intervention**						
Ileocaecal resection	17.9	0.001	48.3	<0.001	39.3	0.2
Subtotal colectomy	27.2	<0.001	5.8	0.003	12.2	0.09
Stoma formation only	19.7	<0.001	1.7	<0.001	4.7	0.2
Others§§	17.9	0.3	22.7	0.4	13.9	0.09
≥ 2 surgical procedures	17.3	0.4	21.5	0.013	29.9	0.14
Laparoscopic surgery	32.4	0.9	32.5	0.051	21.5	0.06
Stoma formation during re-intervention for complications	5.8	0.2	9.9	0.3	9.4	0.9
**Stoma location**						
Ileostomy	81.9	0.051	90.1	0.5	85.1	0.3
Colostomy	14.5	<0.001	1.7	0.08	7.5	0.03
Ileocolostomy	2.9	0.07	7.4	0.4	4.7	0.4
Other	0.6	0.8	0.9	0.13	2.8	0.3
**Stoma configuration**						
Loop/loop-end	45.6	0.006	62.0	0.046	57.9	0.5
End	52.6	0.009	37.2	0.009	36.5	0.9
Other¶¶	1.8	0.5	0.8	0.08	5.6	0.04
**Main procedure—outcomes**						
ICU admission	19.2	0.6	21.7	0.2	26.4	0.4
Duration of in-hospital stay (days), median (i.q.r.)	8.5 (6–15)	0.9	8.0 (6.0–11)	0.6	10 (7.0–15)	0.5
**30-day postoperative complications (Clavien–Dindo)**						
0	44.5	0.5	48.8	0.4	43.0	0.4
I	13.9	0.4	17.4	0.3	19.6	0.7
II	20.8	0.7	19.0	0.10	19.6	0.9
IIIa	8.9	0.3	2.5	0.2	3.7	0.6
IIIb	9.8	0.8	10.7	0.8	14.0	0.4
IV–V	2.9	0.5	1.7	–	0.0	–
Medical complications##	8.7	0.010	1.7	0.2	4.7	0.2
Surgical complications	31.8	0.3	26.5	0.5	28.0	0.8
Required re-operation	11.0	0.9	11.6	0.5	14.0	0.6
30-day mortality rate	1.7	0.15	0.0	–	0.0	–
**Stoma reversal surgery—procedures and outcomes**						
Time between stoma formation and reversal (months), median (i.q.r.)	43 (26–55)		3.0 (2.0–4.0)		10 (7.0–13)	–
Stoma-related overall morbidity rate***	16.8	<0.001	41.3	<0.001	36.5	0.5
**Type of surgery for stoma reversal**						
Open—midline laparotomy	38.5	0.005	23.1	0.8	40.2	0.005
Open—peristomal incision	50.0	0.001	72.7	<0.001	24.2	<0.001
Laparoscopy	11.5	0.025	4.1	0.08	5.6	0.06
**Type of procedure performed**						
Small bowel anastomosis	4.0	0.3	1.7	0.04	6.5	0.06
Ileocolic anastomosis	32.0	0.03	44.6	0.12	51.4	0.30
Colonic anastomosis	8.0	0.014	0.0	0.012	3.7	0.03
Other	56.0	0.7	53.7	0.3	38.3	0.02
New stoma reversal surgery	11.5	<0.001	0.0	0.6	2.9	0.06
Duration of in-hospital stay (days), median (i.q.r.)	6.0 (4.8–8.0)	0.9	5.0 (4.0–6.0)	0.6	6.0 (5.0–10)	0.5
**30-day postoperative complications (Clavien–Dindo)**						
0	64.0	0.10	72.7	0.7	66.4	0.3
I	20.0	0.3	14.9	0.2	10.3	0.3
II	12.0	0.2	7.4	0.5	15.0	0.07
IIIa	0.0	0.9	1.7	0.10	3.7	0.3
IIIb	4.0	0.3	1.7	0.9	3.7	0.3
IV–V	0.0	0.10	1.6	0.2	0.9	0.6
Medical complications	4.0	0.03	0.0	0.5	1.9	0.13
Surgical complications	24.0	0.2	14.1	0.5	19.0	0.3
Required re-operation	4.0	0.5	1.7	0.9	4.7	0.2
30-day mortality rate	3.9	0.03	0.0	0.3	0.9	0.3
6-month mortality rate	1.8	0.14	0.0	0.6	0.9	0.3
18-month mortality rate	3.0	0.06	0.0	0.9	2.8	0.06

Values are % unless otherwise indicated. *Late reversal: reversal more than 18 months after stoma formation. †Chi-squared test for categorical variables; *t*-test and Kruskal–Wallis test for normally distributed and non-normally distributed continuous variables respectively. ‡*P* < 0.050 for no reversal *versus* reversal after 6–18 months. §*P* < 0.050 for 6-month reversal *versus* reversal after 6–18 months. When not reported, *P* values were > 0.050 (indicated as ns). ¶Including: hypertension, cardiovascular disease, pulmonary disease, neurological disease, diabetes, kidney disease, ophthalmologic disease, dermatologic disease, osteoporosis, others. #*P* < 0.050 for no reversal *versus* 6-month reversal. **Including CD with non-stricturing, non-penetrating behaviour, stricturing with perianal disease and other undefined behaviours. ††Including Infliximab, Adalimumab, Vedolizumab, Ustekinumab, others. ‡‡Including abscess, bleeding, fistula, cancer. §§Including redo ileocolic resection, small bowel resection, segmental colectomy. ¶¶Including continent ileostomy (Kock pouch, Barnett continent intestinal reservoir), mucus fistula. ##Including ≥1 among: pulmonary embolism, acute myocardial infarction, prolonged hypertension, prolonged hypotension, pneumothorax, pneumonia, vascular injury due to venous catheter. ***Including ≥1 among: stoma formation-related postoperative complications, 6-month or 18-month stoma-related readmissions, complications due to stoma reversal surgery. ns, not significant; ASA, American Society of Anesthesiologists; s.d., standard deviation; i.q.r., interquartile range; IBD, inflammatory bowel disease; CRP, C-reactive protein; CD, Crohn’s disease.

Predictors for no/late (>18 months) stoma reversal are reported in *Table 4*. In particular, late/non-reversal was not independently associated with age, BMI, smoking, CD location, recurrent CD, previous surgery, preoperative biologic/steroids, preoperative parenteral nutrition, American Society of Anesthesiologists (ASA) score, laparoscopic approach, stoma configuration and stoma-related morbidity rate. Also, penetrating CD (HR 1.36; 95% c.i. 0.95 to 1.95; *P* = 0.090) and ileocaecal resection (HR 1.71; 95% c.i. 0.98 to 2.96; *P* = 0.060) failed to demonstrate an independent association with no/late (>18 months) stoma reversal.

**Table 4:** Multivariate analyses evaluating the potential predictors of no/late (>18 months) stoma reversal during follow-up, in the overall sample and among the subjects with colonic, rectal and perianal CD location only: adjusted hazards ratios (HR) (95% confidence interval (c.i.))

	All CD subjects		Colonic, rectal and perianal CD	
	Adjusted HR (95% c.i.)	P*	Adjusted HR (95% c.i.)	P*
Male sex	0.89 (0.64,1.24)	0.500	0.90 (0.61,1.34)	0.600
Age, 1-year increase	1.00 (0.99,1.02)	0.500	1.00 (0.99,1.02)	0.500
BMI, 1-unit increase	1.02 (0.99,1.04)	0.150	1.01 (0.99,1.04)	0.300
Presence of ≥1 co-morbidity before the start of follow-up, yes *versus* no	0.94 (0.63,1.40)	0.800	0.86 (0.54,1.35)	0.500
Current smoker, yes *versus* no	0.90 (0.59,1.39)	0.600	0.63 (0.36,1.12)	0.110
Colonic, rectal or perianal CD location *versus* others	1.47 (0.91,2.39)	0.120	–	–
Penetrating CD *versus* other behaviours	1.36 (0.95,1.95)	0.090	1.64 (1.10,2.46)	0.016
Previous surgery due to CD, yes *versus* no	0.73 (0.49,1.09)	0.130	0.72 (0.44,1.17)	0.200
Recurrent CD presentation, yes *versus* no	1.32 (0.90,1.95)	0.200	1.32 (0.84,2.09)	0.200
Previous treatment with biologics/steroids *versus* none/other pharmacological treatment†	0.92 (0.58,1.44)	0.700	0.73 (0.41,1.31)	0.300
**ASA score class**				
I (ref. cat.)	–	–	–	–
II	0.52 (0.24,1.12)	0.090	0.74 (0.25,2.19)	0.600
III/IV	0.53 (0.23,1.20)	0.130	0.74 (0.24,2.29)	0.600
Parenteral nutrition preceding surgery, yes *versus* no	1.12 (0.72,1.75)	0.600	1.05 (0.62,1.79)	0.800
Ileocaecal resection *versus* others‡	1.71 (0.98,2.96)	0.060	2.77 (1.32,5.81)	0.007
Laparoscopic *versus* open surgery	0.92 (0.62,1.36)	0.700	1.12 (0.69,1.80)	0.700
End stoma configuration *versus* loop/loop end or others§	0.75(0.52,1.08)	0.120	0.68 (0.43,1.08)	0.100
Stoma-related morbidity, yes *versus* no¶	1.17 (0.75,1.83)	0.500	1.22 (0.71,2.10)	0.500

*Cox proportional hazards analyses including 394 observations and 169 successes (all CD) and 227 observations and 125 successes (colonic, rectal and perianal CD location). †Including antibiotics, azathioprine, mercaptopurine, cyclosporine, thalidomide. ‡Including redo ileocolic resection, small bowel resection, strictureplasty, subtotal colectomy, segmental colectomy, stoma surgery only. §Including continent ileostomy (Kock pouch, Barnett continent intestinal reservoir), mucus fistula. ¶Including ≥1 among: stoma formation-related postoperative complications, 6-month and 18-month stoma-related readmissions. BMI, body mass index; ASA, American Society of Anesthesiologists; CD, Crohn’s disease.

Predictors for the ≤6-month (early) stoma reversal are reported in *Table 5*. In particular, early stoma reversal was positively associated with stoma-related morbidity (HR 1.75; 95% c.i. 1.20 to 2.56; *P* = 0.004) and negatively associated with colonic, rectal or perianal CD location (HR 0.47; 95% c.i. 0.31 to 0.73; *P* = 0.001). No independent association with early stoma reversal was found for age, BMI, smoking, penetrating CD, preoperative biologic/steroids, preoperative parenteral nutrition, ileocaecal resection and stoma configuration.

**Table 5:** Multivariate analyses evaluating the potential predictors of early (within 6 months) stoma reversal during follow-up, in the overall sample and among the subjects with colonic, rectal and perianal CD location only: adjusted hazards ratios (HR) (95% confidence interval (c.i.))

	All patients with CD		Colonic, rectal and perianal CD	
	Adjusted HR (95% c.i.)	P*	Adjusted HR (95% c.i.)	P*
Male sex	0.80 (0.56,1.16)	0.200	0.81 (0.45,1.47)	0.500
Age, 1-year increase	1.00 (0.98,1.01)	0.500	1.00 (0.98,1.02)	0.900
BMI, 1-unit increase	0.99 (0.95,1.03)	0.500	–	–
Current smoker, yes *versus* no	1.17 (0.78,1.74)	0.400	–	–
Colonic, rectal or perianal CD location *versus* others	0.47 (0.31,0.73)	0.001	–	–
Penetrating CD *versus* other behaviours	0.89 (0.61,1.29)	0.500	–	–
Previous treatment with biologics/steroids *versus* none/other pharmacological treatment†	0.78 (0.52,1.15)	0.200	0.53 (0.27,1.04)	0.070
Parenteral nutrition preceding surgery, yes *versus* no	0.80 (0.44,1.47)	0.500	–	–
Ileocaecal resection *versus* others‡	1.47 (0.98,2.20)	0.060	1.92 (0.96,3.86)	0.060
End stoma configuration *versus* loop/loop end or others§	0.80 (0.54,1.18)	0.300	0.40 (0.20,0.78)	0.008
Colostomy, yes *versus* no	1.00 (0.52,1.93)	0.900	–	–
Stoma-related morbidity, yes *versus* no¶	1.75 (1.20,2.56)	0.004	–	–

*Cox proportional hazards analyses including 394 observations and 120 successes (all CD) and 227 observations and 46 successes (colonic, rectal and perianal CD location). †Including antibiotics, azathioprine, mercaptopurine, cyclosporine, thalidomide. ‡Including redo ileocolic resection, small bowel resection, strictureplasty, subtotal colectomy, segmental colectomy, stoma surgery only. §Including continent ileostomy (Kock pouch, Barnett continent intestinal reservoir), mucus fistula. ¶Including ≥1 among: stoma formation-related postoperative complications, 6-month and 18-month stoma-related readmissions. CD, Crohn’s disease.

Morbidity related to stoma presence and reversal was reported as stoma-related overall morbidity, an aggregate outcome including stoma formation-related postoperative complications (*n* = 16 of 401, 3.9%), ≤6-month (*n* = 24 of 401, 6.0%) or 6–18 month (*n* = 7 of 401, 1.5%) stoma-related readmissions and stoma-reversal postoperative complications (*n* = 80 of 401, 20.0%) (*Table 2*). Globally, 118 of 401 (29.4%) patients had stoma-related overall morbidity. Postoperative outcomes after the main procedure with the stoma formation and after stoma reversal surgery are presented overall in *Table 2* according to stoma reversal status at the time of follow-ups in *Table 3*.

The stoma-related overall morbidity rate according to stoma reversal status at the time of follow-up (reversal ≤6 months, reversal 6–18 months and no/late reversal) is reported in *Table 3* with a comparison of clinical and surgical characteristics among the three groups.

Stoma-related morbidity (including stoma formation-related postoperative complications, ≤6-month and 6–18 month stoma-related readmissions) was found to be a predictor for the ≤6-month (early) stoma reversal.

### Subgroup analysis

In a subgroup analysis, 232 (57.9%) patients with colon, rectal and perianal CD were analysed according to primary and secondary outcomes (*Table 6*).

**Table 6:** Colonic, rectal and perianal CD location only: clinical and surgical characteristics of the sample, by time of stoma reversal

	No/late reversal*	*P*	Reversal ≤ 6 months	*P*	Reversal after 6–18 months	*P*†
Variables	(*n* = 127)		(*n* = 46)		(*n* = 59)	
Age (years), mean(s.d.)	43.5(16.8)	0.9	42.2(15.5)	0.6	40.5(15.2)	0.9
Male sex	43.3	0.8	45.7	0.06	61.0	0.12
BMI (kg/m^2^), mean(s.d.)	23.9(7.6)	0.8	23.0(4.5)	0.9	23.6(4.6)	0.9
Familial IBD	7.9	0.3	13.0	0.5	5.1	0.14
At least one risk factor or co-morbidiy‡	40.9	0.07	26.1	0.6	37.3	0.2
**Smoking status**						
Never smoker	59.8	0.2	50.0	0.2	57.6	0.4
Past smoker	18.1	0.9	17.4	0.3	11.9	0.5
Current smoker	22.1	0.14	32.6	0.8	30.5	0.8
Age at IBD diagnosis (years), mean(s.d.)	33.0(16.3)	0.3	28.1(12.9)	0.9	32.8(14.6)	0.4
**CD behaviour**						
Stricturing	32.3	0.9	32.6	0.4	27.1	0.5
Penetrating	50.4	0.2	58.7	0.3	57.6	0.8
Others§	17.3	0.3	8.7	0.7	15.3	0.3
Recurrent CD presentation at the time of surgery	55.1	0.06	39.1	0.4	47.5	0.4
**Medical treatment and laboratory assessment before surgery**						
Previous surgery due to CD	40.2	0.049	24.4	0.09	27.1	0.7
Preoperative medical treatment	92.9	0.5	95.7	0.4	89.5	0.2
	(*n* = 117)		(*n* = 44)		(*n* = 51)	
Treatment with biologics¶	78.6	0.013	59.1	0.10	66.7	0.4
Treatment with steroids	49.6	0.043	31.8	0.020	29.4	0.8
	(*n* = 102)		(*n* = 40)		(*n* = 46)	
Albumin at surgery (g/dl), median (i.q.r.)	3.8 (3.0–23.5)	0.3	3.8 (3.2–4.3)	0.07	3.6 (3.0–4.5)	0.9
	(*n* = 106)		(*n* = 42)		(*n* = 51)	
Hb at surgery (g/dl), median (i.q.r.)	11.3 (10.0–12.4)	0.6	11.7 (10.7–13.1)	0.2	11.5 (10.6–13.0)	0.9
	(*n* = 82)		(*n* = 29)		(*n* = 38)	
CRP at surgery (mg/dl), median (i.q.r.)	8.4 (2.4–31.0)	0.4	7.0 (1.5–42.2)	0.6	6.8 (1.6–15.0)	0.11
**Main procedure—surgical approach**						
**ASA score class at surgery**						
I	3.2	0.3	6.7	0.6	1.7	0.2
II	61.4	0.3	68.9	0.06	74.6	0.5
III	32.3	0.3	24.4	0.3	23.7	0.9
IV	3.1	0.2	0.0	0.2	0.0	–
Emergency surgery	31.5	0.3	23.9	0.9	32.2	0.4
Parenteral nutrition preceding surgery	16.5	0.04	4.4	0.01	3.3	0.8
**Surgery indication**						
Perforation	3.9	0.8	4.4	0.2	8.5	0.06
Obstruction	24.4	0.9	21.7	0.3	17.0	0.5
Persistent inflammation	18.1	0.8	19.6	0.11	8.5	0.09
Others††	22.1	0.9	21.7	0.7	25.3	0.07
≥ 2 coexisting indications	31.5	0.9	32.6	0.2	40.7	0.4
**Type of intervention**						
Ileocaecal resection	7.1	0.3	2.7	0.001	23.7	0.002
Subtotal colectomy	34.7	0.011	15.2	0.04	20.3	0.5
Stoma formation only	22.1	0.007	4.4	0.03	8.5	0.4
Others§§	13.3	<0.001	49.4	0.12	22.1	0.004
≥ 2 surgical procedures	22.8	0.5	28.3	0.8	25.4	0.7
Laparoscopic surgery	32.3	0.2	43.5	0.2	22.0	0.3
Stoma formation during re-intervention for complications	2.4	0.2	6.5	0.2	10.2	0.5
**Stoma location**						
Ileostomy	80.3	0.9	91.3	0.6	83.0	0.2
Colostomy	16.5	0.02	2.2	0.7	13.6	0.03
Ileocolostomy	2.4	0.5	4.4	0.7	3.4	0.8
Other	0.8	0.5	2.1	0.6	0.0	0.3
**Stoma configuration**						
Loop/loop-end	43.7	<0.001	76.1	0.08	57.6	0.044
End	54.8	<0.001	23.9	0.02	37.3	0.2
Other¶¶	1.6	0.6	0.0	0.2	5.1	0.12
**Main procedure—outcomes**						
ICU admission	16.5	0.9	17.4	0.4	22.0	0.6
Duration of in-hospital stay (days), median (i.q.r.)	8.0 (6.0–15)	0.6	8.0 (6.0–10)	0.5	10 (7.0–14)	0.15
**30-day postoperative complications (Clavien–Dindo)**						
0	45.7	0.13	58.7	0.4	39.0	0.04
I	15.8	0.5	19.6	0.4	22.0	0.8
II	21.3	0.6	17.4	0.9	22.0	0.5
IIIa	8.7	0.13	2.2	0.11	1.7	0.9
IIIb	7.1	0.2	2.2	0.11	15.3	0.2
IV–V	1.6	0.4	0.0	0.3	0.0	–
Medical complications##	9.5	0.06	0.0	0.5	6.7	0.07
Surgical complications	29.1	0.06	15.2	0.7	32.2	0.055
Required re-operation	7.1	0.2	2.2	0.2	13.6	0.06
30-day mortality rate	1.6	0.4	0.0	0.3	0.0	–
**Stoma reversal surgery—procedures and outcomes**						
Time between stoma formation and reversal (months), median (i.q.r.)	45 (27–55)		3.0 (2.0–5.0)		11 (7.0–13)	–
Stoma-related overall morbidity rate***	17.3	0.005	37.0	0.001	39.0	0.8
**Type of surgery for stoma reversal**						
Open—midline laparotomy	40.0	<0.001	13.0	0.02	44.1	<0.001
Open—peristomal incision	40.0	<0.001	82.6	0.2	49.1	<0.001
Laparoscopy	20.0	0.011	4.4	0.6	6.8	0.6
**Type of procedure performed**						
Small bowel anastomosis	50.0	0.08	65.2	0.3	40.7	0.015
Ileocolic anastomosis	28.6	0.6	32.6	0.08	42.4	0.3
Colonic anastomosis	7.2	0.2	2.2	0.3	11.9	0.06
Other	14.2	0.007	0.0	0.07	5.0	0.12
New stoma reversal surgery	20.0	0.001	0.0	0.009	3.6	0.14
Duration of in-hospital stay (days), median (i.q.r.)	6.0 (3.0–11)	0.6	5.0 (3.0–6.0)	0.5	6.5 (5.0–11)	0.13
**30-day postoperative complications (Clavien–Dindo)**						
0	57.1	0.006	80.4	0.4	64.4	0.07
I	21.4	0.6	17.4	0.006	5.1	0.04
II	14.3	0.024	2.2	0.2	22.0	0.003
IIIa	0.0	–	0.0	0.14	1.7	0.4
IIIb	7.1	0.07	0.0	0.6	5.1	0.12
IV–V	0.0	–	0.0	0.003	1.7	0.4
Medical complications	7.1	0.06	0.0	0.5	3.4	0.07
Surgical complications	35.7	<0.001	6.5	0.14	25.4	0.012
Required reoperation	7.1	0.07	0.0	0.9	6.7	0.07
30-day mortality rate	0.0	–	0.0	0.6	1.7	0.4
6-month mortality rate	1.6	0.4	0.0	0.3	0.0	–
18-month mortality rate	2.5	0.3	0.0	0.7	3.4	0.2

Values are % unless otherwise indicated. *Late reversal: reversal more than 18 months after stoma formation. †Chi-squared test for categorical variables; *t*-test and Kruskal–Wallis test for normally distributed and non-normally distributed continuous variables respectively. ‡Including: hypertension, cardiovascular disease, pulmonary disease, neurological disease, diabetes, kidney disease, ophthalmologic disease, dermatologic disease, osteoporosis, others. §Including CD with non-stricturing, non-penetrating behaviour, stricturing with perianal disease and other undefined behaviours. ¶Including Infliximab, Adalimumab, Vedolizumab, Ustekinumab, others. #*P* < 0.050 for no reversal *versus* 6-month reversal. **P < 0.050 for no reversal *versus* reversal after 6–18 months. ††Including abscess, bleeding, fistula, cancer. ‡‡*P* < 0.050 for 6-month reversal *versus* reversal after 6–18 months. When not reported, *P* values were > 0.050 (indicated as ns). §§Including redo ileocolic resection, small bowel resection, segmental colectomy. ¶¶Including continent ileostomy (Kock pouch, Barnett continent intestinal reservoir), mucus fistula. ##Including ≥1 among: pulmonary embolism, acute myocardial infarction, prolonged hypertension, prolonged hypotension, pneumothorax, pneumonia, vascular injury due to venous catheter. ***Including ≥1 among: stoma formation-related postoperative complications, 6-month or 18-month readmissions, complications due to stoma reversal surgery. ns, not significant; ASA, American Society of Anesthesiologists; s.d., standard deviation; i.q.r., interquartile range; IBD, inflammatory bowel disease; CRP, C-reactive protein; CD, Crohn’s disease.

At the 6-month follow-up, 46 of 232 (19.8%) patients had the temporary stoma reversed, while 105 of 232 (45.3%) had it reversed at the 18-month follow-up. The major reasons for stoma non-reversal at 6- and 18-month follow-ups were ongoing medical treatment for CD (47.8% and 44.1%), patients unfit for surgery (13.4% and 15.0%), patient’s preference (10.2% and 15.0%) and waiting list (12.1% and 8.1%).

**Fig. 2:**
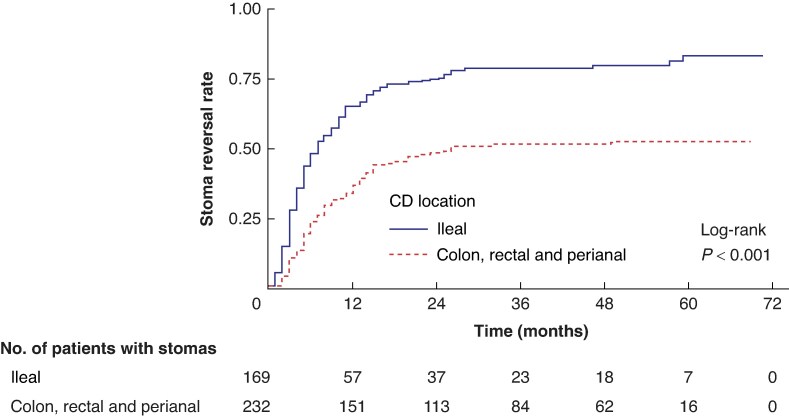
Time-to-event analysis between stoma formation and reversal in patients with colon, rectal and perianal Crohn’s disease (CD) locations compared with ileal bowel location

Time-to-event analysis between stoma formation and reversal in patients with colon, rectal and perianal CD was significantly longer (log-rank *P* < 0.001) compared with ileal localization (*Fig. 2*). Indeed, colon, rectal and perianal CD was found to be an independent predictor for early stoma reversal (HR 0.47; 95% c.i. 0.31 to 0.73; *P* = 0.001) (*Table 5*). However, it was not an independent predictor for no/late stoma reversal (HR 1.47; 95% c.i. 0.91 to 2.39; *P* = 0.120) (*Table 6*).

### COVID-19 impact

**Fig. 3:**
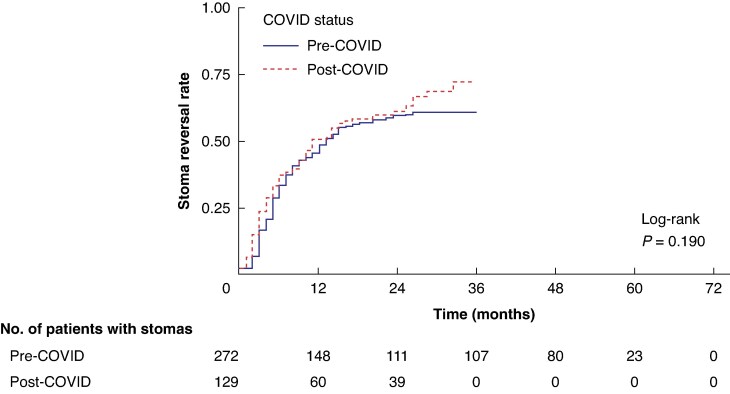
**Time-to-event analysis between stoma formation and reversal in patients with Crohn’s disease (CD) according to the COVID-19 pandemic** Pre-COVID includes patients with CD who underwent stoma formation before October 2019. The post-COVID group included October 2019 and the following months.

The healthcare disruption during the COVID-19 pandemic did not affect the time to stoma reversal surgery (log-rank *P* = 0.190), as shown in *Fig. 3*.

Pre- and post-COVID groups included 272 and 129 patients respectively. The stoma reversal rate at 6- and 18-month follow-ups (primary outcome) was not different between pre- and post-COVID groups. At the 6-month follow-up, the temporary stoma reversal rate was 27.9% (76 of 272) in the pre-COVID *versus* 33.3% (43 of 129) in the post-COVID group (*P* = 0.270); at the 18-month follow-up, it was 154 of 272 (56.6%) *versus* 74 of 129 (57.4%), *P* = 0.890.

## Discussion

This international, multicentre study showed that a large proportion of temporary stomas for CD were not reversed at 6 (69.8%) and 18 months (43.1%) following initial surgery. The most common reasons for non-reversal included ongoing medical treatment, patient not fit for surgery, patient preference and waiting lists. CD patients are exposed to the risk of developing stoma complications for significantly longer intervals and, in some cases, indefinitely.

The stoma-related morbidity rate has been reported to range from 2.9% to 81.1%^8^, with complications such as parastomal hernia, high output stoma, stoma retraction or prolapse. A total of 29.4% of patients had a stoma-related overall morbidity, including postoperative complications (3.9%), readmissions (<6 months 6.0%, 6–18 months 1.5%) and stoma reversal complications (20.0%). Exposing patients to stoma complications long-term should not be overlooked and the risk of non-reversal and associated stoma morbidity should be considered and discussed early on in surgical planning.

Different factors guide the indication for stoma formation, such as emergency surgery, steroid therapy, peritonitis and poor nutritional status. The location of CD also impacts the rate of reversal. In this study, a longer time interval was reported between stoma formation and reversal in a subgroup of patients with perianal, rectal and colonic CD, with 19.8% reversed at 6 months and 45.3% reversed at the 18-month follow-up. These results are reflective of current literature, with a systematic review^9^ reporting 34% of patients undergoing reversal by 6–18-month follow-ups. Of those who underwent reversal, 63% achieved successful restoration of bowel continuity, with the remaining requiring proctectomy or a new stoma formation. The study found only 25% of patients had bowel continuity successfully restored in patients who suffered from refractory perianal or distal colonic CD^9^. The literature also shows the association between perianal CD and permanent stomas, with rates of 30–50% for patients with complex perianal CD ^10,11^ compared with 10% for all CD^12^.

The multidisciplinary management of patients with CD is crucial and requires input from gastroenterologists, surgeons, nutritionists and stoma teams. The role of medical management is to induce and maintain remission. Preoperative optimization and multidisciplinary management aim to improve nutrition and eliminate known risk factors associated with postoperative morbidity and recurrence. Good MDT practice could reduce emergency admissions and urgent surgery rates, reducing stoma formation and the associated morbidity rate^13^. Addressing malnutrition is paramount in managing CD, affecting 65–75% of patients^14^. It is a modifiable risk factor of postoperative complications^15^ with poor nutritional status, described by Caio *et al*.^14^ as a reduction in 10% body weight in the 6 months before surgery, and is associated with poor postoperative outcomes^14,16,17^. Adequate nutrition has not only been shown to reduce complication rates^15^, but a regime of exclusive enteral nutrition before surgery showed 25% of patients with structuring or penetrating CD avoided surgery^18^.

The rates of stoma formation in the biologic era compared with pre-biologics have been investigated with varied results. Some research shows rates of surgery in CD to have reduced over the past six decades^19^ and rates of temporary stomas, which was associated with less emergency surgery^20^. However, the rate of permanent stomas remained static^19^. Other studies show the rate of stoma formation to be static ^12,21^. By working closely with gastroenterologists, patients who are not responding to medical therapies can be flagged to surgical teams earlier and expedited surgery can be planned. It is thought that downgrading surgery from an emergency to a semi-elective setting may in itself reduce the rate of stoma formation.

Patients are counselled before surgery regarding the risk of stoma formation surgery but may not be fully informed about the risk of late or non-reversal and the longer-term morbidity of this. The results of our study could guide discussion on stoma counselling for perianal and colonic CD, which are associated with a longer time interval before reversal, and this cohort of patients should be counselled that there is a higher risk of delayed or permanent stoma. This study has identified factors affecting stoma reversal and aids in personalizing the consenting process to individual patient factors.

Healthcare delivery in CD surgery should also be considered. The CLOSurE of Ileostomy Timing (CLOSE-IT) study showed an 84.9% temporary stoma closure rate after anterior resection for rectal cancer, with a median time to reversal of 259 days^22^. As expected, clinical factors associated with delay differed from those found in CD, including anastomotic leak, cancer progression and chemotherapy. However, aspects of the patient pathway that were associated with delay included outpatient clinic review or imaging before being added to the waiting list for reversal. It is important to highlight that the issue of the waiting list is not applicable to all countries participating in this study as those with private health systems may not have waiting lists. Patients who were added to the waiting lists before outpatient review showed reversals occurred an estimated 133 days sooner than those who were first seen in the clinic and then put on the waiting list for reversal. Developing a standardized treatment pathway for intended temporary stomas could help reduce delays and improve patient experience and quality of life as they are fully informed of the process and steps towards reversal.

This international multicentre study is the first to investigate the reversal rate, timing and complications of intended temporary stomas in CD. A study with a similar aim has been conducted in patients with anastomotic leak after rectal cancer surgery, finding a 1-year stoma-free survival of 45.0%^23^. The large sample size and robust methodology, particularly for data verification and validation, strengthen the reliability of our findings. However, the international, multicentre nature of this study does add some limitations due to the variation in surgical practice across the world. This is an issue considered early on in the study and a survey of surgical practice is being conducted. Our study does not include an evaluation of patient-reported outcomes due to its retrospective nature, and cannot inform discussion on CD recurrence and how stoma formation or late/no reversal affects it. This study was also carried out over a time interval altered by the coronavirus pandemic, with concerns over affecting the study outcomes related to delays in stoma reversal surgery. The impact of this was considered and minimized by carrying out a subgroup analysis that concluded there was no significant difference between pre- and post-coronavirus groups. This article reports stoma non-reversal due to ongoing medical treatments. However, there is no further information on the specific treatments patients were on at that time or what deemed patients as unfit for stoma reversal surgery. This is an area future research could investigate to identify if a particular medical treatment was associated with stoma non-reversal. In addition, the present study is unable to give an indication about the optimal site or type of stoma, or about the optimal circumstances for stoma reversal according to stoma site or type. Indeed, the retrospective design and the sample size do not allow adjustment of the multiple confounders affecting both postoperative outcomes and time of stoma reversal. Therefore, future studies with dedicated design are needed to properly respond to these questions.

In conclusion, this international, multicentre study has shown that a large proportion of intended temporary stomas are not reversed at 6 and 18 months following initial surgery. This has implications on the surgical management of CD and on patient consent. This study highlights the need to shorten the interval between formation and reversal and further investigate how the formation of intended temporary stomas can be reduced.

## Supplementary Material

zraf010_Supplementary_Data

## Data Availability

The anonymized data for this study can be made available upon request.

## Collaborators

### The INTESTINE Study Group

N. Avellaneda 0000-0002-6802-7125 (Nueva Proctología, CEMIC, Buenos Aires, Argentina); A. Potolicchio 0000-0003-0414-8363 (Nueva Proctología, CEMIC, Buenos Aires, Argentina); J. P. Muñoz 0000-0002-4887-1580 (Nueva Proctología, CEMIC, Buenos Aires, Argentina); N. Avellaneda 0000-0002-6802-7125 (Aarhus University Hospital, Aarhus, Denmark); A. S. Abdelrahman 0000-0003-2049-6085 (Giza International Hospital, Giza, Egypt); Sara Mansour Mostafa 0000-0002-6520-1423 (Giza International Hospital, Giza, Egypt); N. de' Angelis 0000-0002-1211-4916 (Beaujon University Hospital (AP-HP), University Paris Cité, Paris, France); C. A. Schena 0000-0003-1136-1103 (Beaujon University Hospital (AP-HP), University Paris Cité, Paris, France); F. Marchegiani 0000-0002-5312-8054 (Beaujon University Hospital (AP-HP), University Paris Cité, Paris, France); M. Kelm 0000-0002-9899-9558 (University Hospital of Wuerzburg, Wuerzburg, Germany); S. Flemming 0000-0002-6304-3169 (University Hospital of Wuerzburg, Wuerzburg, Germany); J. Lock 0000-0002-9007-3937 (University Hospital of Wuerzburg, Wuerzburg, Germany); D. Politis 0000-0001-8520-2071 (Aretaieion Hospital, Athens, Greece); P. Ioannis 0000-0002-4614-9041 (Aretaieion Hospital, Athens, Greece); O. Mangana 0000-0001-8192-8609 (Aretaieion Hospital, Athens, Greece); L. Chardalias 0000-0002-3079-6180 (Aretaieion Hospital, Athens, Greece); Y. Zager 0000-0002-7631-4757 (Sheba Medical Center, Tel Aviv, Israel); N. Horesh 0000-0002-2459-8567 (Sheba Medical Center, Tel Aviv, Israel); G. Calini 0000-0002-7460-9578 (University Hospital of Udine, Udine, Italy); G. Terrosu 0000-0003-2722-7662 (University Hospital of Udine, Udine, Italy); L. Martinuzzo 0000-0002-8706-4773 (University Hospital of Udine, Udine, Italy); D. Muschitiello 0000-0001-8206-7388 (University Hospital of Udine, Udine, Italy); C. Biddau 0000-0002-1225-2921 (Santa Maria degli Angeli Hospital, Pordenone, Italy); A. Braini 0000-0002-1557-4520 (Santa Maria degli Angeli Hospital, Pordenone, Italy); F. Tumminelli 0000-0002-8860-6693 (Santa Maria degli Angeli Hospital, Pordenone, Italy); A. Gori 0000-0002-7780-0618 (IRCCS Azienda Ospedaliero-Universitaria di Bologna, Bologna, Italy); M. Rottoli 0000-0003-0278-4139 (IRCCS Azienda Ospedaliero-Universitaria di Bologna, Bologna, Italy); S. Cardelli 0000-0001-8870-5176 (IRCCS Azienda Ospedaliero-Universitaria di Bologna, Bologna, Italy); A. Belvedere 0000-0003-3662-8273 (IRCCS Azienda Ospedaliero-Universitaria di Bologna, Bologna, Italy); C. Isopi 0000-0002-4186-8952 (IRCCS Azienda Ospedaliero-Universitaria di Bologna, Bologna, Italy); G. Gallo 0000-0003-1066-4671 (Santa Rita Clinic, Vercelli, Italy); M. Trompetto 0000-0003-1066-4671 (Santa Rita Clinic, Vercelli, Italy); G. Clerico 0000-0003-1833-073X (Santa Rita Clinic, Vercelli, Italy); A. Realis Luc 0000-0002-4800-1008 (Santa Rita Clinic, Vercelli, Italy); G. Gallo 0000-0003-1066-4671 (Department of Surgery, Sapienza University of Rome, Rome, Italy); A. Mingoli 0000-0001-5437-9085 (Policlinico Umberto I University Hospital, Sapienza University of Rome, Roma, Italy); P. Lapolla 0000-0002-3088-5263 (Policlinico Umberto I University Hospital, Sapienza University of Rome, Roma, Italy); G. Brachini 0000-0001-6724-5588 (Policlinico Umberto I University Hospital, Sapienza University of Rome, Roma, Italy); G. Mazzarella 0000-0002-1206-9051 (Policlinico Umberto I University Hospital, Sapienza University of Rome, Roma, Italy); O. Ghazouani 0000-0003-1075-0342 (San Paolo Hospital, Savona, Italy); R. Galleano 0000-0001-9787-4908 (San Paolo Hospital, Savona, Italy); M. Malerba 0000-0002-4781-2968 (San Paolo Hospital, Savona, Italy); F. Menegon Tasselli 0000-0002-3611-2763 (Università degli studi della Campania Luigi Vanvitelli, Napoli, Italy); G. Pellino 0000-0002-8322-6421 (Università degli studi della Campania Luigi Vanvitelli, Napoli, Italy); G. Rizzo 0000-0001-9335-6740 (Azienda Ospedaliera Universitaria di Palermo, Palermo, Italy); M. Cappello 0000-0002-9634-4304 (Azienda Ospedaliera Universitaria di Palermo, Palermo, Italy); L. Carrozza 0000-0002-8630-6312 (Azienda Ospedaliera Universitaria di Palermo, Palermo, Italy); G. Mazzarella 0000-0002-1206-9051 (Ospedale San Filippo Neri, Roma, Italy); I. A. Muttillo 0000-0003-0618-4675 (Ospedale San Filippo Neri, Roma, Italy); E. M. Muttillo 0000-0003-3562-0392 (Ospedale San Filippo Neri, Roma, Italy); B. Picardi 0000-0002-1805-5849 (Ospedale San Filippo Neri, Roma, Italy); N. Bazzi 0000-0002-2828-4995 (Al Zahraa Hospital, Beirut, Lebanon); S. Dbouk (Al Zahraa Hospital, Beirut, Lebanon); Z. Chaalan (Al Zahraa Hospital, Beirut, Lebanon); M. Bazzi (Al Zahraa Hospital, Beirut, Lebanon); A. Alkaseek 0000-0002-7915-1826 (Gheryan Central Hospital, Gharyan, Libya); H. Bileid Bakeer 0000-0002-7456-6344 (Gheryan Central Hospital, Gharyan, Libya); H. Shames 0000-0001-9846-5364 (Gheryan Central Hospital, Gharyan, Libya); H. Aboudlal 0000-0001-7624-0332 (Gheryan Central Hospital, Gharyan, Libya); A. Kredan 0000-0002-5558-8118 (Aldiaa Hospital, Tripoli, Libya); Q. Qutaiba 0000-0002-2128-4028 (Al Ahli Hospital, Hebron, Palestine); A. Y. Abu Rumaila 0009-0005-1000-4560 (Al Ahli Hospital, Hebron, Palestine); J. Q. Al Safwan (Qatif Central Hospital, Qatif, Saudi Arabia); N. A. Al Turki (Qatif Central Hospital, Qatif, Saudi Arabia); A. Bunyian (Qatif Central Hospital, Qatif, Saudi Arabia); N. Fernandes Montes 0000-0002-4234-1802 (Vall D'Hebron University Hospital, Barcelona, Spain); M. Martí Gallostra 0000-0002-0783-6359 (Vall D'Hebron University Hospital, Barcelona, Spain); R. Pintos Garza 0000-0002-1225-6666 (Álvaro Cunqueiro Hospital, Vigo, Spain); V. Vigorita 0000-0003-2142-4737 (Álvaro Cunqueiro Hospital, Vigo, Spain); E. Moncada Iribarren 0000-0003-0620-7929 (Álvaro Cunqueiro Hospital, Vigo, Spain); I. De Ariño-Hervas 0000-0002-4343-8243 (Donostia University Hospital, San Sebastián, Spain); I. Aguirre-Allende 0000-0002-5642-860X (Donostia University Hospital, San Sebastián, Spain); J. M. Enriquez-Navascués 0000-0002-6486-1489 (Donostia University Hospital, San Sebastián, Spain); M. J. Padilla-Otamendi 0000-0001-6730-0241 (Donostia University Hospital, San Sebastián, Spain); M. Sanchez-Rodriguez 0000-0002-1630-9956 (University Hospital Gregorio Marañon, Madrid, Spain); C. Pérez-Carpio 0000-0002-7534-1489 (University Hospital Gregorio Marañon, Madrid, Spain); P. Tejedor 0000-0001-8648-7697 (University Hospital Gregorio Marañon, Madrid, Spain); D. Velayos Herraez 0009-0003-2208-0806 (University Hospital Gregorio Marañon, Madrid, Spain); M. D. Cancelas Felgueras 0000-0002-8197-1217 (Hospital Universitario Severo Ochoa, Madrid, Spain); M. Estaire Gómez 0000-0001-9153-9201 (Hospital Universitario Severo Ochoa, Madrid, Spain); E. P. Cagigal Ortega 0000-0003-0668-6376 (Hospital Universitario Severo Ochoa, Madrid, Spain); D. Plazas 0000-0001-9627-154X (La Fe University and Polytechnic Hospital, Valencia, Spain); M. Millan 0000-0001-5321-1432 (La Fe University and Polytechnic Hospital, Valencia, Spain); C. Gutierrez 0009-0003-5331-1304 (La Fe University and Polytechnic Hospital, Valencia, Spain); P. Montalbán 0000-0002-3007-8031 (Hospital Universitario de Salamanca, Salamanca, Spain); F. Blanco-Antona 0000-0001-5946-9944 (Hospital Universitario de Salamanca, Salamanca, Spain); A. E. Valera Montiel 0009-0009-8604-3952 (Hospital Universitario de Salamanca, Salamanca, Spain); R. Kozan 0000-0002-3835-8759 (Gazi Medical School, Ankara, Turkey); S. Leventoglu 0000-0003-0680-0589 (Gazi Medical School, Ankara, Turkey); H. H. Ceylan 0009-0000-5079-5647 (Gazi Medical School, Ankara, Turkey); S. Ozaydin 0000-0002-6406-544X (Gazi Medical School, Ankara, Turkey); E. B. Bostanci 0000-0002-0663-0156 (Ankara City Hospital, Ankara, Turkey); T. Colak 0000-0002-7253-5608 (Mersin University School of Medicine, Sokak, Turkey); C. Ozcan 0000-0002-6453-025X (Mersin University School of Medicine, Sokak, Turkey); I. C. Eray 0000-0002-1560-7740 (Cukurova University School of Medicine, Adana, Turkey); E. Aytaç 0000-0002-8803-0874 (Acıbadem University Faculty of Medicine General Surgery, Istanbul, Turkey); D. Selvakumar 0000-0002-2058-511X (Wythenshawe Hospital, Manchester, UK); L. Hancock 0000-0002-8399-8632 (Wythenshawe Hospital, Manchester, UK); N. Jabble 0000-0002-9713-2696 (Wythenshawe Hospital, Manchester, UK); D. Warrington 0000-0002-0122-2938 (Wythenshawe Hospital, Manchester, UK); N. Ahmed 0000-0002-8976-3578 (Royal Albert Infirmary, Wigan, UK); T. Hussain 0000-0002-7325-2407 (Royal Albert Infirmary, Wigan, UK); J. Cooper 0009-0000-7237-0519 (Royal Albert Infirmary, Wigan, UK); A. Gendia 0000-0001-9351-0664 (Northampton General Hospital, Northampton, UK); J. Ahmed 0000-0001-8058-5480 (Northampton General Hospital, Northampton, UK); K. Exarchou 0000-0003-2057-8232 (Countess of Chester Hospital NHS Foundation Trust, Chester, UK); N. Eardley 0000-0001-6528-4292 (Countess of Chester Hospital NHS Foundation Trust, Chester, UK); B. Davies 0000-0002-9014-183X (Countess of Chester Hospital NHS Foundation Trust, Chester, UK); C. A. Manzo 0000-0002-2401-3434 (Chelsea & Westminster Hospital, London, UK); V. Celentano 0000-0002-3562-9082 (Chelsea & Westminster Hospital, London, UK); S. Pérez-Ajates 0000-0001-9511-1800 (Chelsea & Westminster Hospital, London, UK); S. Seth 0000-0002-2748-8080 (Kingston Hospital, Kingston, UK); K. Sriskandarajah 0000-0003-3359-8961 (Kingston Hospital, Kingston, UK); T. Chouari 0000-0002-1848-5830 (Kingston Hospital, Kingston, UK); E. Matthews 0000-0003-3770-2498 (Royal Devon & Exeter NHS Foundation Trust, Exeter, UK); R. Bethune 0000-0002-1855-0639 (Royal Devon & Exeter NHS Foundation Trust, Exeter, UK); M. Abuelgasim 0000-0002-2906-6711 (Royal Devon & Exeter NHS Foundation Trust, Exeter, UK); A. Smith 0000-0002-6280-566X (Royal Devon & Exeter NHS Foundation Trust, Exeter, UK); E. Brownson 0000-0002-4855-1827 (Queen Elizabeth University Hospital, Glasgow, UK); G. Nicholson 0000-0002-9239-2034 (Queen Elizabeth University Hospital, Glasgow, UK); I. Campbell 0000-0002-8421-9178 (Queen Elizabeth University Hospital, Glasgow, UK); A. Subramanian 0000-0003-1619-8202 (Royal Sussex County Hospital, Brighton, UK); A. Tonsi 0000-0003-0753-899X (Royal Sussex County Hospital, Brighton, UK); J. Siby 0000-0001-5690-4760 (Royal Sussex County Hospital, Brighton, UK); Z. Garoufalia 0000-0002-1766-9777 (Cleveland Clinic Florida, Weston, FL, USA); S. D. Wexner 0000-0001-8046-5753 (Cleveland Clinic Florida, Weston, FL, USA); P. Zhou 0000-0002-2651-0506 (Cleveland Clinic Florida, Weston, FL, USA); R. Gefen 0000-0002-8501-131X (Cleveland Clinic Florida, Weston, FL, USA); M. Arjonilla 0009-0001-0020-501X (Stony Brook University, Stony Brook, NY, USA); F. Monzur 0000-0002-9786-8310 (Stony Brook University, Stony Brook, NY, USA); M. Al-Sadawi 0000-0003-3064-7887 (Stony Brook University, Stony Brook, NY, USA).

### The INTESTINE Study Group - National Coordinators

France: N. de’ Angelis 0000-0002-1211-4916 (Beaujon University Hospital (AP-HP), Paris, France).

Greece: O. Mangana 0000-0001-8192-8609 (Aretaieion Hospital, Athens, Greece).

Israel: N. Horesh 0000-0002-2459-8567 (Sheba Medical Center, Tel Aviv, Israel).

Italy: G. Calini 0000-0002-7460-9578 (University Hospital of Udine, Udine, Italy).

Middle East and North Africa: M. Elhadi 0000-0001-6406-4212 (Tripoli University Hospital, Tripoli, Libya).

Spain: P. Tejedor 0000-0001-8648-7697 (University Hospital Gregorio Marañon, Madrid, Spain); V. Vigorita 0000-0003-2142-4737 (Álvaro Cunqueiro Hospital, Vigo, Spain).

Turkey: S. Leventoglu 0000-0003-0680-0589 (Gazi Medical School, Ankara, Turkey).

United Kingdom (UK): C. Perrott 0000-0002-2039-6511 (Chelsea & Westminster Hospital, London, UK).

United States of America (USA): Z. Garoufalia 0000-0002-1766-9777 (Cleveland Clinic Florida, Weston, FL, USA).

### The INTESTINE Study Group - Steering Group

Principal Investigator: V. Celentano 0000-0002-3562-9082 (Chelsea & Westminster Hospital, London, UK).

Associate Principal Investigators: C. Perrott 0000-0002-2039-6511 (Chelsea & Westminster Hospital, London, UK); M. Rottoli 0000-0003-0278-4139 (IRCCS Azienda Ospedaliero-Universitaria di Bologna, Bologna, Italy).

Corresponding Authors: V. Celentano 0000-0002-3562-9082 (Chelsea & Westminster Hospital, London, UK); G. Calini 0000-0002-7460-9578 (University Hospital of Udine, Udine, Italy).

Methodological Leads: G. Calini 0000-0002-7460-9578 (University Hospital of Udine, Udine, Italy); C. Kontovounisios 0000-0002-1828-1395 (The Royal Marsden NHS Foundation Trust, London, UK).

Supervisors: Z. Garoufalia 0000-0002-1766-9777 (Cleveland Clinic Florida, Weston, FL, USA); M. Elhadi 0000-0001-6406-4212 (Tripoli University Hospital, Tripoli, Libya); S. D. Wexner 0000-0001-8046-5753 (Cleveland Clinic Florida, Weston, FL, USA).

Patient representative: S. Blackwell 0000-0002-2819-3727 (Patient representative, Liverpool, UK).

Trainee leaders: C. Perrot 0000-0002-2039-651 (Chelsea & Westminster Hospital, London, UK); G. Calini 0000-0002-7460-9578 (University Hospital of Udine, Udine, Italy); A. Gori 0000-0002-7780-0618 (IRCCS Azienda Ospedaliero-Universitaria di Bologna, Bologna, Italy).
