# Supplementary material for: Outcomes of intended temporary stomas in Crohn's disease (INTESTINE study): international, multicentre, retrospective study
Source: BJS Open. 2025 Jun 2;9(3):zraf010. doi: 10.1093/bjsopen/zraf010 (PMC12128195; doi:10.1093/bjsopen/zraf010)
Supplement: zraf010_Supplementary_Data [file zraf010_supplementary_data.zip › Supplementary -The INTESTINE Study.docx]

**The INTESTINE Study: an international, multicenter, retrospective study on outcomes of INtendedTEmporarySTomas In crohN’s disease.**

Claire Perrott BMBS, MSc* ^1^, Giacomo Calini M.D.* ^2,3,4^, Alice Gori M.D. ^3,4^, Matteo Rottoli, M.D., PhD,^3,4^, Maria E. Flacco M.D. ^5^, Lamberto Manzoli M.D ^4^., Zoe Garoufalia M.D.^6^, Steven D. Wexner M.D., PhD,^6^, Christos Kontovounisios M.D., PhD, FACS, FRCS^1,7,8,9^, Muhammed Elhadi MBBCh, MSc^10^,V. Celentano M.D., PhD, ^1,9^,The INTESTINE study group.

* equally contributed

^1^ Chelsea and Westminster Hospital NHS Foundation Trust, London, UK

^2^ ClinicaChirurgica, University Hospital of Udine, Udine, Italy

^3^ Surgery of the AlimentaryTract, IRCCS Azienda Ospedaliero-Universitaria di Bologna, Bologna, Italy

^4^ Department of Medical and Surgical Sciences, Alma Mater Studiorum - University of Bologna, Bologna, Italy

^5^ Department of Environmental and Preventive Sciences, University of Ferrara, Ferrara, Italy

^6^ Cleveland Clinic Florida, Ellen Leifer Shulman and Steven Shulman Digestive Disease Center, Weston, United States

^7^ 2^nd^ Surgical Department, Evaggelismos Athens General Hospital, Athens, Greece

^8^ The Royal Marsden NHS Foundation Trust, London, UK

^9^ Department of Surgery and Cancer, Imperial College, London, UK

^10^ Tripoli University Hospital, Tripoli, Libya.

Collaborators of *The INTESTINE study group* are listed in the Appendix 1

**Correspondence**

Valerio Celentano, MD, PhD, FRCS

Consultant Colorectal Surgeon

Inflammatory Bowel Disease and Ileoanal Pouch Surgery Centre, Chelsea and Westminster Hospital NHS Foundation Trust,369 Fulham Road, London, UK.

Department of Surgery and Cancer, Imperial College, London, UK

valerio.celentano@nhs.net
https://orcid.org/0000-0002-3562-9082

X: @VCsurgery

Giacomo Calini, MD

Consultant Surgeon, Surgery of the AlimentaryTract, IRCCS Azienda Ospedaliero-Universitaria di Bologna, Italy

Assistant Professor of Surgery (RTDa), Dept. of Medical and Surgical Sciences, Alma Mater Studiorum - University of Bologna, Italy

giacomo.calini2@unibo.it
https://orcid.org/0000-0002-7460-9578

X: @GCalini

**Supplementary Materials - Index**

| **Supplementary Figures and Tables** |  |
| --- | --- |
| Figure S1. The INTESTINE Study Timeline | *Page 4* |
| Figure S2. Flowchart of data verification and validation  Figure S3. The INTESTINE Study – Geographical distribution of participating centers | *Page 5*  *Page 6* |

**Figure S1.**


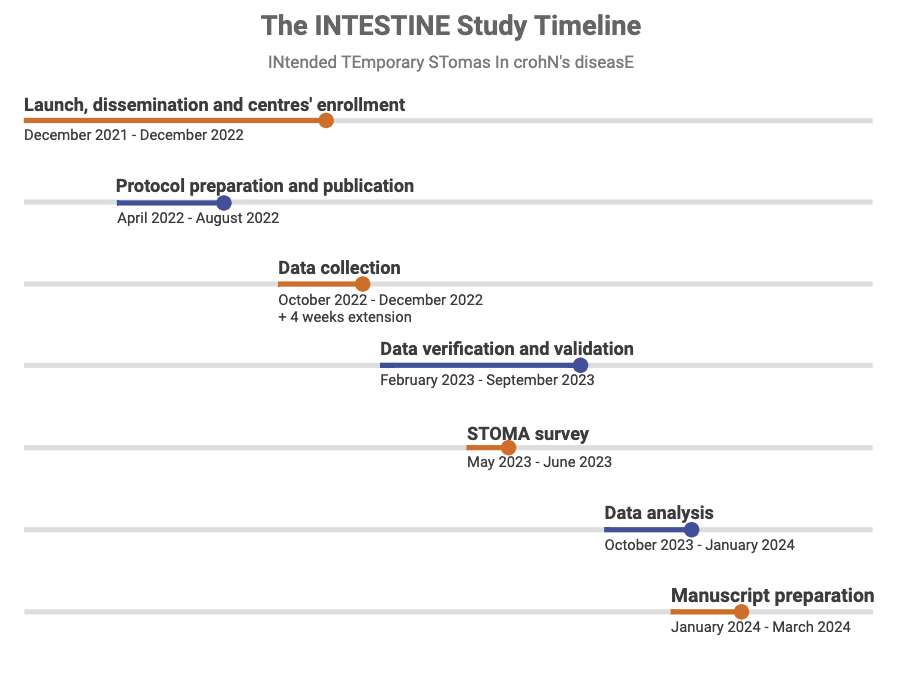
created with BioRender.com

**Figure S2. Flowchart of data verification and validation**

**
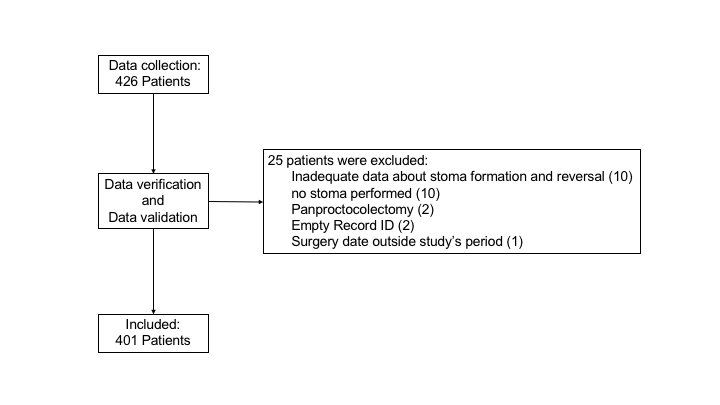
**

**Figure S3.**


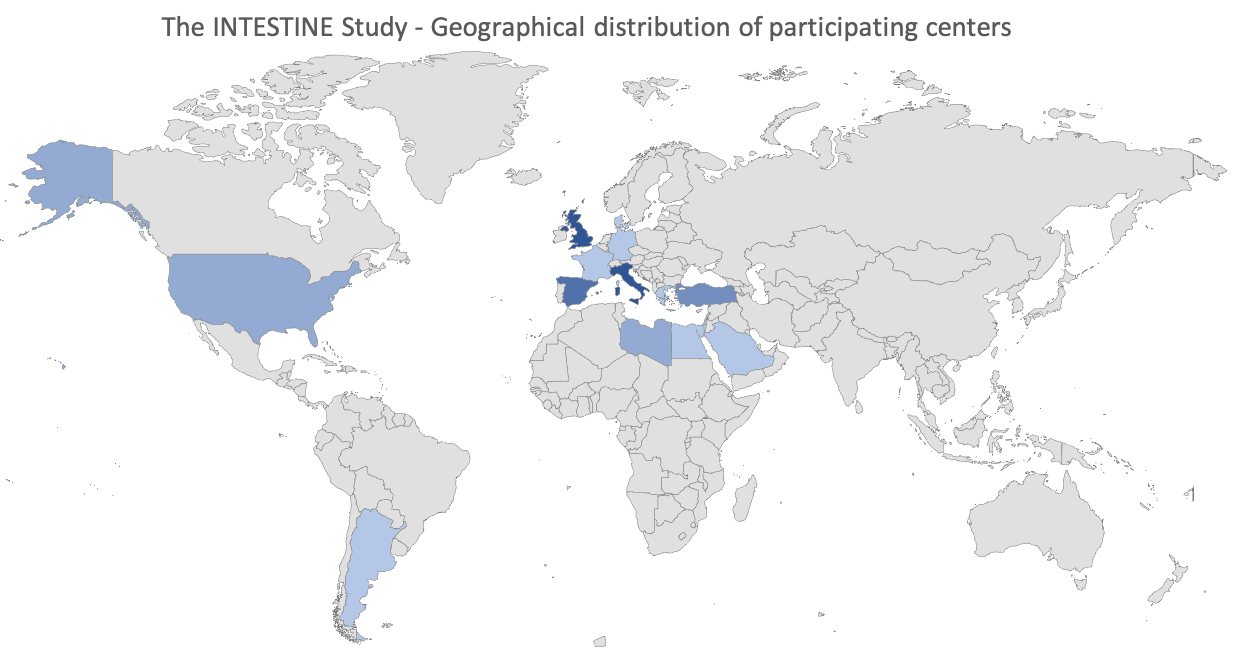


Number of participant centers: 44. Argentina (1), Denmark (1), Egypt (1), France (1), Germany (1), Greece (1), Israel (1), Italy (9), Lebanon (1), Libya (2), Palestine (1), Saudi Arabia (1), Spain (7), Turkey (5), United Kingdom (9), United States of America (2).
