# Supplementary material for: Outcomes of intended temporary stomas in Crohn's disease (INTESTINE study): international, multicentre, retrospective study
Source: BJS Open. 2025 Jun 2;9(3):zraf010. doi: 10.1093/bjsopen/zraf010 (PMC12128195; doi:10.1093/bjsopen/zraf010)
Supplement: zraf010_Supplementary_Data [file zraf010_supplementary_data.zip › The INTESTINE Study Group - Collaborative Authors.docx]

**The INTESTINE Study Group - Collaborators**
ORCID id (0000-0000-0000-0000) is indicated when available

N. Avellaneda 0000-0002-6802-7125 (Nueva Proctología, CEMIC, Buenos Aires, Argentina); A. Potolicchio 0000-0003-0414-8363 (Nueva Proctología, CEMIC, Buenos Aires, Argentina; J. P. Muñoz 0000-0002-4887-1580 (Nueva Proctología, CEMIC, Buenos Aires, Argentina); N. Avellaneda 0000-0002-6802-7125 (Aarhus University Hospital, Aarhus, Denmark); A. S. Abdelrahman 0000-0003-2049-6085 (Giza International Hospital, Giza, Egypt); Sara Mansour Mostafa 0000-0002-6520-1423 (Giza International Hospital, Giza, Egypt); N. de' Angelis 0000-0002-1211-4916 (Beaujon University Hospital (AP-HP), University Paris Cité, Paris, France); C. A. Schena 0000-0003-1136-1103 (Beaujon University Hospital (AP-HP), University Paris Cité, Paris, France); F. Marchegiani 0000-0002-5312-8054 (Beaujon University Hospital (AP-HP), University Paris Cité, Paris, France); M. Kelm 0000-0002-9899-9558 (University Hospital of Wuerzburg, Wuerzburg, Germany); S. Flemming 0000-0002-6304-3169 (University Hospital of Wuerzburg, Wuerzburg, Germany); J. Lock 0000-0002-9007-3937 (University Hospital of Wuerzburg, Wuerzburg, Germany); D. Politis 0000-0001-8520-2071 (Aretaieion Hospital, Athens, Greece); P. Ioannis  0000-0002-4614-9041 (Aretaieion Hospital, Athens, Greece); O. Mangana 0000-0001-8192-8609 (Aretaieion Hospital, Athens, Greece); L. Chardalias 0000-0002-3079-6180 (Aretaieion Hospital, Athens, Greece); Y. Zager 0000-0002-7631-4757 (Sheba Medical Center, Tel Aviv, Israel); N. Horesh 0000-0002-2459-8567 (Sheba Medical Center, Tel Aviv, Israel); G. Calini 0000-0002-7460-9578 (University Hospital of Udine, Udine, Italy); G. Terrosu 0000-0003-2722-7662 (University Hospital of Udine, Udine, Italy); L. Martinuzzo 0000-0002-8706-4773 (University Hospital of Udine, Udine, Italy); D. Muschitiello 0000-0001-8206-7388 (University Hospital of Udine, Udine, Italy); C. Biddau 0000-0002-1225-2921 (Santa Maria degli Angeli Hospital, Pordenone, Italy); A. Braini 0000-0002-1557-4520 (Santa Maria degli Angeli Hospital, Pordenone, Italy); F. Tumminelli 0000-0002-8860-6693 (Santa Maria degli Angeli Hospital, Pordenone, Italy); A. Gori 0000-0002-7780-0618 (IRCCS Azienda Ospedaliero-Universitaria di Bologna, Bologna, Italy); M. Rottoli 0000-0003-0278-4139 (IRCCS Azienda Ospedaliero-Universitaria di Bologna, Bologna, Italy); S. Cardelli 0000-0001-8870-5176 (IRCCS Azienda Ospedaliero-Universitaria di Bologna, Bologna, Italy); A. Belvedere 0000-0003-3662-8273 (IRCCS Azienda Ospedaliero-Universitaria di Bologna, Bologna, Italy); C. Isopi 0000-0002-4186-8952 (IRCCS Azienda Ospedaliero-Universitaria di Bologna, Bologna, Italy); G. Gallo 0000-0003-1066-4671 (Santa Rita Clinic, Vercelli, Italy); M. Trompetto 0000-0003-1066-4671 (Santa Rita Clinic, Vercelli, Italy); G. Clerico 0000-0003-1833-073X (Santa Rita Clinic, Vercelli, Italy); A. Realis Luc 0000-0002-4800-1008 (Santa Rita Clinic, Vercelli, Italy); G. Gallo 0000-0003-1066-4671 (Department of Surgery, Sapienza University of Rome, Rome, Italy); A. Mingoli 0000-0001-5437-9085 (Policlinico Umberto I University Hospital, Sapienza University of Rome, Roma, Italy); P. Lapolla 0000-0002-3088-5263 (Policlinico Umberto I University Hospital, Sapienza University of Rome, Roma, Italy); G. Brachini 0000-0001-6724-5588 (Policlinico Umberto I University Hospital, Sapienza University of Rome, Roma, Italy); G. Mazzarella 0000-0002-1206-9051 (Policlinico Umberto I University Hospital, Sapienza University of Rome, Roma, Italy); O. Ghazouani 0000-0003-1075-0342 (San Paolo Hospital, Savona, Italy); R. Galleano 0000-0001-9787-4908 (San Paolo Hospital, Savona, Italy); M. Malerba 0000-0002-4781-2968 (San Paolo Hospital, Savona, Italy); F. Menegon Tasselli 0000-0002-3611-2763 (Università degli studi della Campania Luigi Vanvitelli, Napoli, Italy); G. Pellino 0000-0002-8322-6421 (Università degli studi della Campania Luigi Vanvitelli, Napoli, Italy); G. Rizzo 0000-0001-9335-6740 (Azienda Ospedaliera Universitaria di Palermo, Palermo, Italy); M. Cappello 0000-0002-9634-4304 (Azienda Ospedaliera Universitaria di Palermo, Palermo, Italy); L. Carrozza 0000-0002-8630-6312 (Azienda Ospedaliera Universitaria di Palermo, Palermo, Italy); G. Mazzarella 0000-0002-1206-9051 (Ospedale San Filippo Neri, Roma, Italy); I. A. Muttillo 0000-0003-0618-4675 (Ospedale San Filippo Neri, Roma, Italy); E. M. Muttillo 0000-0003-3562-0392 (Ospedale San Filippo Neri, Roma, Italy); B. Picardi 0000-0002-1805-5849 (Ospedale San Filippo Neri, Roma, Italy); N. Bazzi 0000-0002-2828-4995 (Al Zahraa Hospital, Beirut, Lebanon); S. Dbouk (Al Zahraa Hospital, Beirut, Lebanon); Z. Chaalan (Al Zahraa Hospital, Beirut, Lebanon); M. Bazzi (Al Zahraa Hospital, Beirut, Lebanon); A. Alkaseek, 0000-0002-7915-1826 (Gheryan Central Hospital, Gharyan, Libya); H. Bileid Bakeer 0000-0002-7456-6344 (Gheryan Central Hospital, Gharyan, Libya); H. Shames 0000-0001-9846-5364 (Gheryan Central Hospital, Gharyan, Libya); H. Aboudlal 0000-0001-7624-0332 (Gheryan Central Hospital, Gharyan, Libya); A. Kredan, 0000-0002-5558-8118 (Aldiaa Hospital, Tripoli, Libya); Q. Qutaiba 0000-0002-2128-4028(Al Ahli hospital, Hebron, Palestine); A. Y. Abu Rumaila 0009-0005-1000-4560 (Al Ahli hospital, Hebron, Palestine); J. Q. Al Safwan (Qatif central hospital, Qatif, Saudi Arabia); N. A. Al Turki (Qatif central hospital, Qatif, Saudi Arabia); A. Bunyian (Qatif central hospital, Qatif, Saudi Arabia); N. Fernandes Montes 0000-0002-4234-1802 (Vall D’Hebron University Hospital, Barcelona, Spain); M. Martí Gallostra 0000-0002-0783-6359 (Vall D’Hebron University Hospital, Barcelona, Spain); R. Pintos Garza 0000-0002-1225-6666 (Álvaro Cunqueiro Hospital, Vigo, Spain); V. Vigorita 0000-0003-2142-4737 (Álvaro Cunqueiro Hospital, Vigo, Spain); E. Moncada Iribarren 0000-0003-0620-7929 (Álvaro Cunqueiro Hospital, Vigo, Spain); I. De Ariño-Hervas 0000-0002-4343-8243 (Donostia University Hospital, San Sebastián, Spain); I. Aguirre-Allende 0000-0002-5642-860X (Donostia University Hospital, San Sebastián, Spain); J. M. Enriquez-Navascués 0000-0002-6486-1489 (Donostia University Hospital, San Sebastián, Spain); M. J. Padilla-Otamendi 0000-0001-6730-0241 (Donostia University Hospital, San Sebastián, Spain); M. Sanchez-Rodriguez 0000-0002-1630-9956 (University Hospital Gregorio Marañon, Madrid, Spain); C. Pérez-Carpio 0000-0002-7534-1489 (University Hospital Gregorio Marañon, Madrid, Spain); P. Tejedor 0000-0001-8648-7697 (University Hospital Gregorio Marañon, Madrid, Spain); D. Velayos Herraez 0009-0003-2208-0806 (University Hospital Gregorio Marañon, Madrid, Spain); M. D. Cancelas Felgueras 0000-0002-8197-1217 (Hospital Universitario Severo Ochoa, Madrid, Spain); M. Estaire Gómez 0000-0001-9153-9201 (Hospital Universitario Severo Ochoa, Madrid, Spain); E. P. Cagigal Ortega 0000-0003-0668-6376 (Hospital Universitario Severo Ochoa, Madrid, Spain); D. Plazas 0000-0001-9627-154X (La Fe University and Polytechnic Hospital, Valencia, Spain); M. Millan 0000-0001-5321-1432 (La Fe University and Polytechnic Hospital, Valencia, Spain); C. Gutierrez 0009-0003-5331-1304 (La Fe University and Polytechnic Hospital, Valencia, Spain); P. Montalbán 0000-0002-3007-8031 (Hospital Universitario de Salamanca, Salamanca, Spain); F. Blanco-Antona 0000-0001-5946-9944 (Hospital Universitario de Salamanca, Salamanca, Spain); A. E. Valera Montiel 0009-0009-8604-3952 (Hospital Universitario de Salamanca, Salamanca, Spain); R. Kozan 0000-0002-3835-8759 (Gazi Medical School, Ankara, Turkey); S. Leventoglu 0000-0003-0680-0589 (Gazi Medical School, Ankara, Turkey); H. H. Ceylan 0009-0000-5079-5647 (Gazi Medical School, Ankara, Turkey); S. Ozaydin 0000-0002-6406-544X (Gazi Medical School, Ankara, Turkey); E. B. Bostanci 0000-0002-0663-0156 (Ankara City Hospital, Ankara, Turkey); T. Colak 0000-0002-7253-5608 (Mersin University School of Medicine, Sokak, Turkey); C. Ozcan 000-0002-6453-025X (Mersin University School of Medicine, Sokak, Turkey); I. C. Eray 0000-0002-1560-7740 (Cukurova University School of Medicine, Adana, Turkey); E. Aytaç 0000-0002-8803-0874 (Acıbadem University Faculty of Medicine General Surgery, Istanbul, Turkey); D. Selvakumar 0000-0002-2058-511X (Wythenshawe Hospital, Manchester, UK); L. Hancock 0000-0002-8399-8632 (Wythenshawe Hospital, Manchester, UK); N. Jabble 0000-0002-9713-2696 (Wythenshawe Hospital, Manchester, UK); D. Warrington 0000-0002-0122-2938 (Wythenshawe Hospital, Manchester, UK); N. Ahmed 0000-0002-8976-3578 (Royal Albert Infirmary, Wigan, UK); T. Hussain 0000-0002-7325-2407 (Royal Albert Infirmary, Wigan, UK); J. Cooper 0009-0000-7237-0519 (Royal Albert Infirmary, Wigan, UK); A. Gendia 0000-0001-9351-0664 (Northampton General Hospital, Northampton, UK); J. Ahmed 0000-0001-8058-5480 (Northampton General Hospital, Northampton, UK); K. Exarchou 0000-0003-2057-8232 (Countess of Chester Hospital NHS Foundation Trust, Chester, UK); N. Eardley 0000-0001-6528-4292 (Countess of Chester Hospital NHS Foundation Trust, Chester, UK); B. Davies 0000-0002-9014-183X (Countess of Chester Hospital NHS Foundation Trust, Chester, UK); C. A. Manzo 0000-0002-2401-3434 (Chelsea & Westminster Hospital, London, UK); V. Celentano 0000-0002-3562-9082 (Chelsea & Westminster Hospital, London, UK); S. Pérez-Ajates 0000-0001-9511-1800 (Chelsea & Westminster Hospital, London, UK); S. Seth 0000-0002-2748-8080 (Kingston Hospital, Kingston, UK); K. Sriskandarajah 0000-0003-3359-8961 (Kingston Hospital, Kingston, UK); T. Chouari 0000-0002-1848-5830 (Kingston Hospital, Kingston, UK); E. Matthews 0000-0003-3770-2498 (Royal Devon & Exeter NHS Foundation Trust, Exeter, UK); R. Bethune 0000-0002-1855-0639 (Royal Devon & Exeter NHS Foundation Trust, Exeter, UK); M. Abuelgasim 0000-0002-2906-6711 (Royal Devon & Exeter NHS Foundation Trust, Exeter, UK); A. Smith 0000-0002-6280-566X (Royal Devon & Exeter NHS Foundation Trust, Exeter, UK); E. Brownson 0000-0002-4855-1827 (Queen Elizabeth University Hospital, Glasgow, UK); G. Nicholson 0000-0002-9239-2034 (Queen Elizabeth University Hospital, Glasgow, UK); I. Campbell 0000-0002-8421-9178 (Queen Elizabeth University Hospital, Glasgow, UK); A. Subramanian 0000-0003-1619-8202 (Royal Sussex County Hospital, Brighton, UK); A. Tonsi 0000-0003-0753-899X (Royal Sussex County Hospital, Brighton, UK); J. Siby 0000-0001-5690-4760 (Royal Sussex County Hospital, Brighton, UK); Z. Garoufalia 0000-0002-1766-9777 (Cleveland Clinic Florida, Weston, FL, USA); S. D. Wexner 0000-0001-8046-5753 (Cleveland Clinic Florida, Weston, FL, USA); P. Zhou 0000-0002-2651-0506 (Cleveland Clinic Florida, Weston, FL, USA); R. Gefen 0000-0002-8501-131X (Cleveland Clinic Florida, Weston, FL, USA); M. Arjonilla 0009-0001-0020-501X (Stony Brook University, Stony Brook, NY, USA); F. Monzur 0000-0002-9786-8310 (Stony Brook University, Stony Brook, NY, USA); M. Al-Sadawi 0000-0003-3064-7887 (Stony Brook University, Stony Brook, NY, USA);

**The INTESTINE Study Group - National Coordinators**

France

N. de’ Angelis 0000-0002-1211-4916 (Beaujon University Hospital (AP-HP), Paris, France)

Greece

O. Mangana 0000-0001-8192-8609 (Aretaieion Hospital, Athens, Greece)

Israel

N. Horesh 0000-0002-2459-8567 (Sheba Medical Center, Tel Aviv, Israel)

Italy

G. Calini 0000-0002-7460-9578 (University Hospital of Udine, Udine, Italy)

Middle East and North Africa

M. Elhadi 0000-0001-6406-4212 (Tripoli University Hospital, Tripoli, Libya)

Spain

P. Tejedor 0000-0001-8648-7697 (University Hospital Gregorio Marañon, Madrid, Spain); V. Vigorita 0000-0003-2142-4737 (Álvaro Cunqueiro Hospital, Vigo, Spain)

Turkey

S. Leventoglu 0000-0003-0680-0589 (Gazi Medical School, Ankara, Turkey)

United Kingdom (UK)

C. Perrott 0000-0002-2039-6511 (Chelsea & Westminster Hospital, London, UK)

United States of America (USA)

Z. Garoufalia 0000-0002-1766-9777 (Cleveland Clinic Florida, Weston, FL, USA)

**The INTESTINE Study Group - Steering Group**

ORCID id (0000-0000-0000-0000) is indicated.

Principal Investigator

V. Celentano 0000-0002-3562-9082 (Chelsea & Westminster Hospital, London, UK)

Associate Principal Investigators

C. Perrott 0000-0002-2039-6511 (Chelsea & Westminster Hospital, London, UK); M. Rottoli 0000-0003-0278-4139 (IRCCS Azienda Ospedaliero-Universitaria di Bologna, Bologna, Italy).

Corresponding Authors

V. Celentano 0000-0002-3562-9082 (Chelsea & Westminster Hospital, London, UK); G. Calini 0000-0002-7460-9578 (University Hospital of Udine, Udine, Italy).

Methodological Leads

G. Calini 0000-0002-7460-9578 (University Hospital of Udine, Udine, Italy); C. Kontovounisios 0000-0002-1828-1395 (The Royal Marsden NHS Foundation Trust, London, UK).

Supervisors

Z. Garoufalia 0000-0002-1766-9777 (Cleveland Clinic Florida, Weston, FL, USA); M. Elhadi 0000-0001-6406-4212 (Tripoli University Hospital, Tripoli, Libya); S. D. Wexner 0000-0001-8046-5753 (Cleveland Clinic Florida, Weston, FL, USA).

Patient representative

S. Blackwell 0000-0002-2819-3727 (Patient representative, Liverpool, UK).

Trainee leaders

C. Perrot 0000-0002-2039-651 (Chelsea & Westminster Hospital, London, UK); G. Calini 0000-0002-7460-9578 (University Hospital of Udine, Udine, Italy); A. Gori 0000-0002-7780-0618 (IRCCS Azienda Ospedaliero-Universitaria di Bologna, Bologna, Italy).
